# Association between serum 25-hydroxyvitamin D concentrations and overactive bladder in female: a mediation analysis of inflammatory biomarkers

**DOI:** 10.3389/fnut.2026.1741019

**Published:** 2026-06-09

**Authors:** Tong Liu, Shaofan An, Jiayi Liu, Peng Zhang

**Affiliations:** 1 The First Affiliated Hospital of Dalian Medical University, Dalian, China; 2 Department of Urology, The First Affiliated Hospital of Dalian Medical University, Dalian, China

**Keywords:** inflammatory biomarkers, mediation analysis, multi-cohort study, overactive bladder, serum 25-hydroxyvitamin D

## Abstract

**Background:**

Overactive bladder (OAB) is a prevalent condition that substantially impairs quality of life. Although 25-hydroxyvitamin D [25(OH)D] plays an important role in immune modulation, its association with OAB and the potential contribution of inflammation remain incompletely understood.

**Methods:**

We analyzed 25,096 participants from National Health and Nutrition Examination Survey (NHANES) 2007–2018 and 2,442 participants from a hospital-based replication cohort (FAH-DMU-DB) using multivariable logistic regression and restricted cubic spline models. Inflammatory biomarkers were incorporated to evaluate their influence on the association between 25(OH)D and OAB. A two-sample Mendelian randomization (MR) analysis was additionally performed to assess potential relationship.

**Results:**

In the NHANES cohort, higher serum 25(OH)D levels were inversely associated with the risk of OAB among females but not males. Female in the highest quartile of 25(OH)D exhibited a significantly lower risk of OAB compared with those in the lowest quartile (OR = 0.733, 95% CI: 0.589–0.912, *p* = 0.005). Consistent findings were observed in the FAH-DMU-DB cohort, where serum 25(OH)D levels were significantly associated with decreased risk of OAB (OR = 0.490, 95% CI: 0.245–0.978, *p =* 0.043). Inflammatory biomarkers, particularly the systemic immune-inflammation index and neutrophil count, attenuated the protective association between 25(OH)D and OAB. MR analysis provided additional support for the observed association, showing that genetically predicted higher vitamin D levels were associated with a reduced risk of urinary incontinence/bladder problem (inverse variance weighted: both *p* < 0.05).

**Conclusion:**

Higher serum 25(OH)D levels are associated with a lower prevalence of OAB in females. Concordant observational and genetic evidence suggests a potential protective role of vitamin D, with systemic inflammation potentially modulating this association.

## 1 Introduction

Overactive bladder (OAB) is a common urological disorder characterized by urinary urgency, typically with increased frequency and nocturia, with or without urge urinary incontinence (UUI) (1, 2). These symptoms can significantly impair quality of life, particularly in aging populations (3). Although antimuscarinic agents have remained the cornerstone of treatment, their effectiveness is often limited by partial symptom relief, side effects, and declining efficacy over time due to tolerance (4). These limitations have prompted growing interest in preventive strategies aimed at slowing symptom progression and reducing long-term reliance on medication.

The precise etiology of OAB remains incompletely elucidated, with multiple overlapping mechanisms likely contributing to symptom development. Among the earliest proposed explanations is the myogenic hypothesis, which attributes urinary urgency and frequency to abnormal spontaneous contractions of the detrusor muscle. However, accumulating evidence now highlights a significant role for immune-inflammatory mechanisms in OAB. Clinical studies have demonstrated elevated circulating levels of C-reactive protein (CRP) in individuals with OAB, suggesting the presence of chronic bladder inflammation (5). Furthermore, increased concentrations of serum adipokines—key mediators involved in metabolic regulation and inflammation—have been observed in OAB patients, supporting a potential link between systemic inflammatory status and bladder dysfunction (6, 7). These findings point to inflammation as a contributing factor in OAB and provide a rationale for further investigation into its role in disease onset and progression.

Furthermore, 25-hydroxyvitamin D [25(OH)D], the primary storage form of vitamin D, has gained attention as a potential factor associated with bladder dysfunction, owing to its broad biological roles in both muscle physiology and immune regulation (8). Acting through its receptor, 25(OH)D contributes to the maintenance of skeletal and smooth muscle integrity, including detrusor muscle function, and is involved in the modulation of inflammatory response (2, 9). These physiological effects have led to increasing interest in the possible association between serum 25(OH)D status and OAB. Several observational studies have reported a decreased level of 25(OH)D in individuals with OAB, and meta-analyses have suggested a potential link between lower serum 25(OH)D levels and increased OAB risk (10, 11). Nonetheless, findings remain inconsistent across studies, with some studies, particularly those focused on female populations, have found no significant association (4). To date, large-scale, population-based investigation specifically addressing the relationship between serum 25(OH)D concentrations and OAB incidence is lacking.

In this study, we investigated the association between 25(OH)D concentrations and the incidence of OAB using data derived from the United States National Health and Nutrition Examination Survey (NHANES) and a hospital-based replication cohort from the First Affiliated Hospital of Dalian Medical University Database (FAH-DMU-DB). By leveraging publicly available population-based data, our goal was to provide epidemiological evidence that could inform future clinical guidelines for OAB management. In addition, we explored the role of systemic inflammation in the association between serum 25(OH)D levels and OAB. To further provide complementary evidence, a two-sample Mendelian randomization analysis was conducted to evaluate the potential association.

## 2 Method

### 2.1 Study population

Data for the present study were obtained from two sources. The first source was the NHANES, developed by the National Center for Health Statistics (NCHS) in the United States. NHANES is a nationally representative, cross-sectional survey that collects comprehensive information through interviews, physical examinations, and laboratory tests, employing a stratified, multistage probability sampling design to generate critical health statistics. Ethical oversight of the survey is provided by the NCHS Institutional Review Board, with written informed consent obtained from all participants.

**Figure 1:**
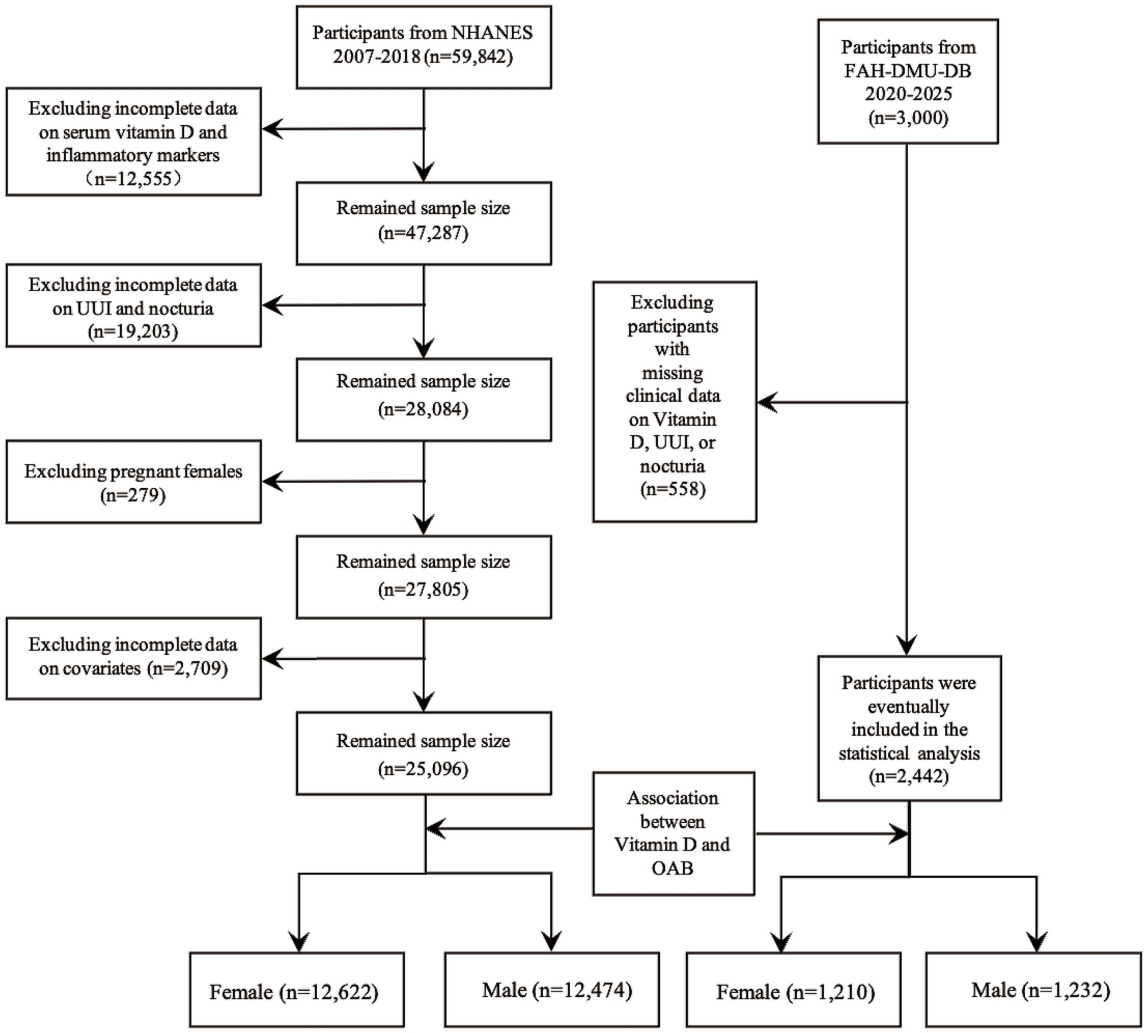
Overview of study design and participants excluded from the study.

We utilized data from six consecutive NHANES cycles (2007–2008, 2009–2010, 2011–2012, 2013–2014, 2015–2016, and 2017–2018) for this study. Of the original 59,842 participants, exclusion criteria were systematically applied to remove 12,555 individuals with incomplete serum 25(OH)D and inflammatory biomarkers evaluations, 19,203 lacking complete information on UUI and nocturia, 279 pregnant women, and 2,709 participants with missing covariate data. After applying these criteria, a total of 25,096 participants were included in the final analysis (Figure 1).

To replicate the main findings in a specific clinical setting, we utilized data from the FAH-DMU-DB, covering the period from January 2020 to January 2025. Data were extracted from the Electronic Medical Records (EMR) system of adult outpatients attending the Department of Urology, including both first-time consultations and follow-up visits, primarily for the evaluation of lower urinary tract symptoms or routine urological assessment.

Unlike the population-based NHANES cohort, FAH-DMU-DB represents a hospital-based, referral clinical population enriched for urinary symptoms and therefore at higher risk of OAB. Among the 3,000 initially screened outpatients, 558 individuals were excluded due to incomplete 25(OH)D and inflammatory biomarker measurements, missing OAB-related diagnostic information, or unavailable covariate data. The final analytical sample consisted of 2,442 participants (Figure 1). This study component was approved by the hospital’s Ethics Committee and was conducted in accordance with the Declaration of Helsinki.

### 2.2 Measurement of serum 25(OH)D

To ensure methodological consistency, we restricted our analyses to NHANES 2007–2018, during which serum 25(OH)D was uniformly measured using a standardized method. Specifically, serum levels of 25(OH)D were measured using a fully validated and standardized liquid chromatography–tandem mass spectrometry (LC–MS/MS) method for all eligible participants (12, 13).

### 2.3 OAB diagnosis

In the NHANES cohot, OAB was defined according to the International Continence Society (ICS) as: urinary urgency, usually accompanied by frequency and nocturia, with or without UUI, in the absence of urinary tract infection or other obvious pathology. Accordingly, we utilized the Overactive Bladder Symptom Score (OABSS), a well-validated and widely used questionnaire, to assess OAB (14). The OABSS evaluates symptom severity primarily through assessments of UUI and nocturia. UUI was determined by two survey questions: (1) “During the past 12 months, have you leaked or lost control of even a small amount of urine with an urge or pressure to urinate and you could not get to the toilet fast enough?” and (2) “How frequently does this occur?” Nocturia was assessed with the question: “During the past 30 days, how many times per night did you most typically get up to urinate, from the time you went to bed at night until the time you got up in the morning? (15, 16)” Participants were classified as having OAB if they had a total OABSS of ≥3 (17). Symptom severity was further categorized based on the OABSS into three levels: mild (score = 3), moderate (score = 4), and severe (score = 5–6), as detailed in Supplementary Figure S1.

In FAH-DMU-DB, OAB was ascertained based on clinician diagnosis recorded in the EMR. Specifically, patients were classified as having OAB if they had a documented diagnosis corresponding to International Classification of Diseases, 10th Revision (ICD-10) code N32.81 (Overactive bladder). Diagnoses were made by board-certified urologists following routine clinical evaluation in a tertiary care setting. Consistent with this referral-based design, the prevalence of OAB in FAH-DMU-DB was substantially higher than that observed in NHANES, reflecting a selected clinical population rather than the general population.

### 2.4 Measurement of inflammatory biomarkers

According to the NHANES 2007–2018 protocol, complete blood cell counts were obtained from peripheral blood samples collected at the Mobile Examination Center (MEC) using the Beckman Coulter method, which combines cell counting and sizing with an automated diluting and mixing system, along with a single-beam photometer for hemoglobin measurement. In this study, the assessed inflammatory biomarkers included white blood cell (WBC) count, neutrophil count, neutrophil-to-lymphocyte ratio (NLR), and systemic immune-inflammation index (SII). Lymphocyte, neutrophil, and platelet counts were expressed in units of 10^9^ cells/L. SII was calculated as: platelet count × neutrophil count / lymphocyte count (7, 18, 19).

### 2.5 Covariates

The following baseline covariates were included: age (<50, 50–75, and ≥75 years old), sex (male or female), race/ethnicity (White, Black, Mexican, Other), family income-to-poverty ratio (PIR) (≤1.3, 1.3–3.5, >3.5), educational attainment (below than high school, high school graduate, above high school), and marital status (married or never married), season of blood collection. Information on hypertension and diabetes was based on participants’ self-reports, including whether they had ever been diagnosed or were currently taking antihypertensive or glucose-lowering medications, since medication use might normalize clinical measurements. Smoking status was classified as never, former, or current smoker. Alcohol use was defined as consuming at least 12 drinks in the past year.

Vitamin D supplementation was assessed using one or two 24-h dietary recall interviews. If both were available, we used the average dose; otherwise, the single reported dose was used. Obesity was indexed using BMI. Height, weight, and waist circumference were measured following NHANES protocols. Besides, physical activity data came from the Global Physical Activity Questionnaire (GPAQ), covering activity during work, transportation, and leisure. Following WHO guidelines, weekly activity was expressed in metabolic equivalent (MET) minutes, derived from the type, frequency, and duration of reported activities (20, 21).

Several chronic diseases as covariates in this study were defined as follows: asthma was identified through self-reported physician diagnosis. Chronic obstructive pulmonary disease (COPD) was defined by a combination of post-bronchodilator FEV1/FVC < 0.7, reported diagnoses of emphysema or chronic bronchitis, and/or current use of medications. Cardiovascular disease (CVD) was also assessed by questionnaire; participants were considered to have CVD if they reported a physician diagnosis of heart attack, coronary heart disease, angina, heart failure, or stroke. Responses marked as “do not know” were excluded.

### 2.6 Mendelian randomization analysis

MR analyses were conducted to investigate the potential association between circulating vitamin D levels and bladder health outcomes using a two-sample MR design. Publicly available, female-specific genome-wide association study (GWAS) summary statistics were obtained from the UK Biobank (Neale Lab Round 2 analysis: https://www.nealelab.is/uk-biobank). Genetic instruments for the exposure factor vitamin D level were derived from a GWAS of female participants of European ancestry in the UK Biobank (Code: 30890_irnt). Bladder health outcomes were assessed using GWAS summary statistics from the same source, including unspecified urinary incontinence (Code: R32) and bladder problem (Code: 20002_1201). Detailed information is available in Supplementary Table S1. Single-nucleotide polymorphisms (SNPs) associated with vitamin D levels at genome-wide significance (*p* < 1 × 10^−5^) were selected as candidate instrumental variables. To ensure the independence of the genetic instruments, linkage disequilibrium (LD) clumping was performed using a European reference panel, applying a threshold of r^2^ < 0.001 within a 1-Mb window. Exposure and outcome datasets were subsequently harmonized to align effect alleles across datasets. Palindromic SNPs with intermediate allele frequencies were excluded to avoid strand ambiguity, and SNPs unavailable in the outcome datasets were removed. After quality control and harmonization, a total of 118 independent SNPs were retained for the final MR analyses (Supplementary Table S2).

The primary causal estimates were obtained using the inverse variance–weighted (IVW) method under a random-effects model. Sensitivity analyses were performed using MR-Egger regression, weighted median, and weighted mode approaches to evaluate the robustness of the findings under different assumptions regarding horizontal pleiotropy. Directional pleiotropy was assessed using the MR-Egger intercept test, while heterogeneity among SNP-specific causal estimates was examined using Cochran’s Q statistic. To further assess the stability and reliability of the MR results, standard diagnostic visualizations were generated, including scatter plots of SNP-specific exposure–outcome associations, leave-one-out analyses to evaluate the influence of individual SNPs on the overall estimates, and funnel plots to visually inspect symmetry and potential pleiotropic effects (Supplementary Figure S2). Detailed information on all instrumental SNPs, including rsIDs, effect alleles, effect sizes, standard errors, and F-statistics, is provided in the Supplementary Table S11.

### 2.7 Statistical analysis

According to the NHANES analytic protocol, all datasets were integrated into a single analytic file, and all statistical analyses accounted for the masked variance structure and applied the recommended survey weighting methodology. Sample weights derived from the Mobile Examination Center (MEC) component were used to address nonresponse, noncoverage, and unequal probabilities of selection. To combine six consecutive NHANES cycles spanning 12 years (2007–2018), MEC examination weights were reweighted in accordance with NHANES analytic guidelines and prior studies. Specifically, the full sample 2-year MEC examination weights (WTMEC2YR) were divided by six to generate combined survey weights, as follows:

$$\begin{array}{l} WT_{2007–2018}=\frac{1}{6}\times \text{WTMEC}2YR_{2007–2008}\\ +\frac{1}{6}\times \text{WTMEC}2YR_{2009–2010}\\ +\frac{1}{6}\times \text{WTMEC}2YR_{2011–2012}\\ +\frac{1}{6}\times \text{WTMEC}2YR_{2013–2014}\\ +\frac{1}{6}\times \text{WTMEC}2YR_{2015–2016}\\ +\frac{1}{6}\times \text{WTMEC}2YR_{2017–2018}. \end{array}$$

All analyses incorporated the combined MEC examination weights along with the NHANES-provided stratification (SDMVSTRA) and primary sampling unit (SDMVPSU) variables to appropriately account for the complex, multistage sampling design.

Furthermore, baseline characteristics of the study population were summarized using survey-weighted means ± standard deviation (SD) for continuous variables and survey-weighted frequencies (percentages) for categorical variables. Differences in continuous variables were assessed using weighted linear regression or the Wilcoxon rank-sum test, depending on the normality determined by the Lilliefors test. Categorical variables were compared using the weighted chi-square test. Given the non-normal distribution of serum 25(OH)D, log-transformed values (log₁₀[25(OH)D]) were utilized for continuous analysis in logistic regression models. However, to ensure clinical relevance, raw values were used for categorical analyses and Restricted Cubic Spline (RCS) visualizations to accurately depict dose–response associations (7). Non-linear associations between 25(OH)D levels and OAB risk were examined using RCS (22). Multivariable logistic regression models were constructed incorporating sample weights, clustering, and stratification to estimate odds ratios (ORs) and 95% confidence intervals (CIs), utilizing the ‘survey’ package in R to account for the complex NHANES survey design.

To ensure a scientifically rigorous selection of covariates, we first employed a directed acyclic graph (DAG) framework to pre-identify variables with plausible relationships to both serum 25(OH)D levels and OAB, while minimizing the inclusion of variables that may introduce collider or mediator bias. Following this, we used variance inflation factor (VIF) analysis to assess multicollinearity among candidate covariates (Supplementary Figure S3). Variables with high collinearity (VIF > 5) were excluded from the final models to preserve model interpretability. Based on this approach, Model 2 was adjusted for a set of established demographic confounders: age, race, marital status, PIR, and education level. Model 3 further incorporated lifestyle and clinical variables that may mediate or confound the vitamin D–OAB relationship, including smoking, drinking, hypertension, diabetes, BMI, asthma, COPD, CVD, SII, vitamin D supplement use, season of blood collection and physical activity MET. Median analyses were conducted using the Mediation package of R software to evaluated whether inflammatory markers mediated the relationship between 25(OH)D and OAB (23). The total effect was decomposed into direct and indirect effects; the indirect effect represented the pathway mediated by inflammatory markers, and the proportion mediated indicated the percentage of the total effect attributable to mediation.

Subgroup analyses and interaction tests were conducted for exploratory purposes; therefore, no statistical adjustment for multiple comparisons was applied. To assess the potential impact of missing covariate data on the robustness of our findings, we conducted sensitivity analyses using multiple imputation. Missing covariate values were imputed using multivariate imputation by chained equations under the assumption that data were missing at random. Five imputed datasets were generated, and parameter estimates were combined using Rubin’s rules. All statistical analyses were conducted using R software version 4.4.3, with the aid of the “stats 4.4.3,” “mgcv 1.9.1” “car 3.1.2” “olsrr 0.5.4” “mediation 4.5.1” “dplyr 1.1.4” “semPlot 1.1.6” “tibble 3.2.1” “ggplot2 3.5.1” “dagitty 0.3.1” “bnlearn 4.9” and “lavaan 0.6.19” packages, and a two-tailed *p* value <0.05 was considered statistically significant.

## 3 Results

### 3.1 Baseline characteristics of participants

A total of 25,096 participants from NHANES 2007–2018 (Table 1) and 2,442 participants from the FAH-DMU-DB cohort were included (Supplementary Table S3). In NHANES, participants with higher serum 25(OH)D levels were older, had lower BMI, and exhibited more favorable socioeconomic and lifestyle profiles. Mean age increased from 44.9 ± 16.8 to 55.7 ± 17.5 years, while BMI decreased from 32.3 ± 9.6 to 28.1 ± 6.3 kg/m^2^ (*p* < 0.001). Higher 25(OH)D levels were associated with being White, married, better educated, and having higher income (*p* < 0.001). Vitamin D supplement use rose from 6.7 to 58.4% across increasing categories. The prevalence of OAB declined from 25.6 to 18.9% (*p* < 0.001), and participants with higher 25(OH)D had lower SII, neutrophil counts, and NLR values, indicating reduced systemic inflammation.

**Table 1:** Baseline characteristics of NHANES participants between 2007 and 2018.

Variables	25(OH)D (nmol/L)				*p* value
	<25; Weighted *N* = 5,159,365; Unweighted *n* = 1,120	25–49.9; Weighted *N* = 37,029,086; Unweighted *n* = 6,755	50–74.9; Weighted *N* = 66,294,880; Unweighted *n* = 9,177	≥75; Weighted *N* = 70,169,163; Unweighted *n* = 8,044	
N	1,120	6,755	9,177	8,044	
Age (years, mean ± SD)	44.94 ± 16.79	45.54 ± 16.72	48.71 ± 17.02	55.73 ± 17.48	<0.001
Gender (%)					<0.001
Male	497 (44.28)	3,397 (50.29)	4,994 (54.42)	3,586 (44.58)	
Female	623 (55.72)	3,358 (49.72)	4,183 (45.58)	4,458 (55.42)	
Race (%)					<0.001
White	136 (12.14)	1,562 (23.12)	4,107 (44.75)	5,188 (64.49)	
Black	638 (56.96)	2,236 (33.10)	1,329 (14.48)	883 (10.98)	
Mexican	158 (14.11)	1,350 (19.99)	1,562 (17.02)	576 (7.16)	
Other	188 (16.79)	1,607 (23.79)	2,179 (23.75)	1,397 (17.37)	
Marital status (%)					<0.001
Never married	610 (54.46)	3,068 (45.42)	3,463 (37.74)	3,016 (37.49)	
Married	510 (45.54)	3,687 (54.58)	5,714 (62.26)	5,028 (62.51)	
Educational levels (%)					<0.001
< High school	73 (6.52)	672 (9.94)	1,005 (10.95)	589 (7.32)	
High school	483 (43.13)	2,697 (39.92)	3,302 (35.98)	2,672 (33.22)	
> High school	564 (50.35)	3,386 (50.14)	4,870 (53.07)	4,783 (59.46)	
Poverty ratio (%)					<0.001
< 1.3	434 (38.75)	2,517 (37.26)	2,910 (31.72)	2,016 (25.06)	
1.3–3.5	469 (41.88)	2,634 (38.99)	3,508 (38.22)	2,909 (36.16)	
> 3.5	217 (19.37)	1,604 (23.75)	2,759 (30.06)	3,119 (38.78)	
Seasonality(%)					<0.001
November–March	753 (67.23)	3,897 (57.69)	4,196 (45.72)	3,097 (38.50)	
April–October	367 (32.77)	2,858 (42.31)	4,981 (54.28)	4,947 (61.50)	
Vitamin D supplement use (%) < 0.001					
No	1,045 (93.30)	5,948 (88.05)	6,523 (71.08)	3,345 (41.58)	
Yes	75 (6.70)	807 (11.95)	2,654 (28.92)	4,699 (58.42)	
Diabetes (%)					<0.001
No	947 (84.55)	5,820 (86.15)	7,948 (86.61)	6,760 (84.03)	
Yes	173 (15.45)	935 (13.85)	1,229 (13.39)	1,284 (15.97)	
Hypertension (%)					<0.001
No	706 (63.04)	4,462 (66.05)	6,111 (66.59)	4,507 (56.03)	
Yes	414 (36.96)	2,293 (33.95)	3,066 (33.41)	3,537 (43.97)	
Drinking (%)					<0.001
No	472 (42.14)	2,712 (40.15)	3,433 (37.41)	3,177 (39.49)	
Yes	648 (57.86)	4,043 (59.85)	5,744 (62.59)	4,867 (60.51)	
Smoking (%)					<0.001
Never	179 (15.98)	1,288 (19.07)	2,317 (25.25)	2,429 (30.20)	
Former	324 (28.93)	1,577 (23.35)	1,843 (20.08)	1,348 (16.76)	
Now	617 (55.09)	3,890 (57.59)	5,017 (54.67)	4,267 (53.05)	
OAB (%)					<0.001
No	833 (74.38)	5,380 (79.65)	7,434 (81.01)	6,222 (77.35)	
Yes	287 (25.62)	1,375 (20.35)	1,743 (18.99)	1,822 (22.65)	
OABSS (%)					<0.001
None OABSS<3	833 (74.38)	5,380 (79.65)	7,434 (81.01)	6,222 (77.35)	
Low OABSS = 3	170 (15.18)	885 (13.10)	1,132 (12.34)	1,132 (14.07)	
Median OABSS = 4	75 (6.70)	309 (4.57)	390 (4.25)	439 (5.46)	
High OABSS = 5, 6	42 (3.74)	181 (2.68)	221 (2.40)	251 (3.12)	
Asthma (%)					0.031
No	931 (83.13)	5,714 (84.59)	7,878 (85.85)	6,860 (85.28)	
Yes	189 (16.87)	1,041 (15.41)	1,299 (14.15)	1,184 (14.72)	
COPD (%)					<0.001
No	1,022 (91.25)	6,254 (92.58)	8,503 (92.66)	7,275 (90.44)	
Yes	98 (8.75)	501 (7.42)	674 (7.34)	769 (9.56)	
CVD (%)					<0.001
No	1,077 (96.16)	6,542 (96.85)	8,921 (97.21)	7,737 (96.18)	
Yes	43 (3.84)	213 (3.15)	256 (2.79)	307 (3.82)	
BMI (kg/m^2^, mean ± SD)	32.34 ± 9.58	30.48 ± 7.58	29.28 ± 6.61	28.07 ± 6.28	<0.001
Inflammatory biomarkers (mean ± SD) < 0.001					
SII	517.01 ± 332.47	517.27 ± 468.18	517.28 ± 318.22	539.89 ± 351.81	
Neutrophils	4.11 ± 1.76	4.25 ± 2.13	4.25 ± 1.62	4.24 ± 1.66	
WBC	7.13 ± 2.22	7.32 ± 5.42	7.26 ± 2.21	7.18 ± 2.86	
NLR	2.01 ± 1.12	2.08 ± 1.17	2.13 ± 1.12	2.27 ± 1.30	
Physical activity MET (mean ± SD)					
	3,036.51 ± 5,978.62	3,625.64 ± 6,346.12	3,853.31 ± 6,371.22	3,474.07 ± 5,882.016	<0.001

Mean ± SD for continuous variables, and p-value was calculated by weighted t-test. %, categorical variables, and *p*-value was calculated by weighted χ^2^ test. SII, Systemic immune-inflammation index; WBC, White blood cell; NLR, neutrophil-to-lymphocyte ratio; PIR, poverty income ratio; OAB, Overactive bladder; OABSS, Overactive bladder symptom score. Analyses followed the NHANES complex survey design and weighting recommendations issued by the National Center for Health Statistics.

In the FAH-DMU-DB cohort, participants were generally older (mean age 65.7 ± 19.4 years) and all of Chinese Han ethnicity (Supplementary Table S3). A similar pattern was observed: higher serum 25(OH)D levels were linked to lower inflammatory indices and slightly lower OAB prevalence (from 88.4 to 74.9%, *p* < 0.001; Supplementary Table S3). Overall, across both populations, higher serum 25(OH)D levels were consistently associated with older age, lower BMI, healthier lifestyles, and lower systemic inflammation, supporting a protective association between vitamin D and OAB risk.

### 3.2 Association between serum 25(OH)D levels and OAB

In the NHANES cohort, the association between serum 25(OH)D levels and OAB was evaluated using multivariable logistic regression analysis. In the unadjusted model, serum 25(OH)D levels were positively associated with OAB in the total population (OR: 1.201, 95% CI: 1.029–1.403, *p* = 0.020; Table 2). However, after adjusting for demographic and clinical covariates, this association reversed, indicating a protective effect in the fully adjusted model (Model 3: OR: 0.800, 95% CI: 0.667–0.958, *p* = 0.016; Table 2). When 25(OH)D concentrations were analyzed as categorical variables using <25 nmol/L as the reference, a consistent inverse association with OAB was observed across all models in the total population. In Model 3, individuals with serum 25(OH)D levels of 25–49.9, 50–74.9, and ≥75 nmol/L had significantly reduced odds of OAB (ORs: 0.847 [95% CI: 0.720–0.997, *p* = 0.046]; 0.815 [95% CI: 0.692–0.960, *p* = 0.015]; and 0.769 [95% CI: 0.650–0.909, *p* = 0.003], respectively; Table 2).

**Table 2:** Association between serum 25(OH)D concentration and OAB.

Variables (%)	Model1 OR (95%CI)	Model2 OR (95%CI)	Model3 OR (95%CI)
Total			
Log_10_(25(OH)D)	1.201 (1.029,1.403) 0.020	0.790 (0.660,0.944) 0.009	0.800 (0.667,0.958) 0.016
<25	1.0	1.0	1.0
25–49.9	0.742 (0.641,0.859) 0.001	0.827 (0.704,0.970) 0.020	0.847 (0.720,0.997) 0.046
50–74.9	0.680 (0.589,0.786) < 0.001	0.775 (0.660,0.910) 0.001	0.815 (0.692,0.960) 0.015
>75	0.850 (0.736,0.982) 0.026	0.751 (0.637,0.885) 0.001	0.769 (0.650,0.909) 0.003
Female			
Log_10_(25(OH)D)	0.909 (0.749,1.103)0.333	0.716 (0.572,0.896) 0.003	0.770 (0.601,0.987) 0.040
<25	1.0	1.0	1.0
25–49.9	0.725 (0.602,0.874) 0.001	0.812 (0.664,0.994) 0.043	0.825 (0.671,1.014) 0.068
50–74.9	0.643 (0.535,0.774) < 0.001	0.742 (0.605,0.909) 0.004	0.771 (0.625,0.951) 0.015
>75	0.730 (0.608,0.876) 0.001	0.701 (0.570,0.862) 0.001	0.733 (0.589,0.912) 0.005
Male			
Log_10_(25(OH)D)	1.753 (1.354,2.270) 0.001	0.976 (0.727,1.311) 0.872	1.013 (0.736,1.395) 0.940
<25	1.0	1.0	1.0
25–49.9	0.832 (0.652,1.061) 0.137	0.859 (0.659,1.120) 0.260	0.893 (0.682,1.169) 0.409
50–74.9	0.829 (0.654,1.052) 0.122	0.826 (0.634,1.077) 0.158	0.904 (0.689,1.184) 0.462
>75	1.085 (0.853,1.379) 0.505	0.848 (0.646,1.112) 0.232	0.890 (0.671,1.180) 0.683

OR, Odds ratio; CI, Confidence interval. Model 1: no covariates were adjusted. Model 2: age, race, marital status, poverty income ratio, and education level were adjusted. Model 3: age, race, marital status, poverty income ratio, education level, smoking, drinking, hypertension, diabetes, BMI, asthma, COPD, CVS, SII, vitamin D supplement use, season of blood collection, and physical activity MET were adjuste.

Sex-stratified analyses revealed a significant inverse association between 25(OH)D levels and OAB among females (Table 2). In the fully adjusted model, higher (25(OH)D) levels were associated with a lower risk of OAB (OR: 0.770, 95% CI: 0.601–0.987, *p* = 0.040). Furthermore, compared with those with vitamin D < 25 nmol/L, female with levels of 50–74.9 nmol/L and ≥75 nmol/L had significantly lower odds of OAB in the fully adjusted model (OR: 0.771, 95% CI: 0.625–0.951, *p* = 0.015; OR: 0.733, 95% CI: 0.589–0.912, *p* = 0.005). In contrast, no statistically significant associations were identified between 25(OH)D levels and OAB risk in male participants across all models (Table 2). These findings suggest that higher serum 25(OH)D levels are independently associated with a lower prevalence of OAB, particularly in female. To assess the consistency of results, we performed a sensitivity analysis using multiple imputation for missing covariates. The results corroborated the primary analysis, indicating that higher serum 25(OH)D levels were significantly associated with a decreased OAB risk in both the overall population and the female subgroup (Supplementary Table S4). In a sensitivity analysis using a more stringent OAB definition (OABSS ≥ 4) in NHANES, the inverse association between serum 25(OH)D levels and OAB remained materially unchanged in females, with similar effect estimates observed (Supplementary Table S5).

In the FAH-DMU-DB, consistent protective associations were observed. Higher serum 25(OH)D levels were significantly associated with a reduced risk of OAB (Model 1: OR = 0.516, 95% CI: 0.363–0.733; Model 2: OR = 0.465, 95% CI: 0.310–0.699; *p* < 0.001; Supplementary Table S6). Compared with <25 nmol/L, higher 25(OH)D levels (25–49.9, 50–74.9, and ≥75 nmol/L) were associated with progressively lower odds of OAB in the adjusted model (OR = 0.777, 0.698, and 0.600, respectively; Supplementary Table S6). Sex-stratified analyses in the FAH-DMU-DB cohort showed that the protective association was significant among females (Model 2: OR = 0.373, 95% CI: 0.216–0.643, *p* < 0.001; Supplementary Table S6). Compared with <25 nmol/L, females with serum 25(OH)D levels of 50–74.9 and ≥75 nmol/L had significantly reduced odds of OAB (OR = 0.573 [95% CI: 0.369–0.890, *p* = 0.013]; OR = 0.490 [95% CI: 0.245–0.978, *p* = 0.043]). No significant association was observed among males (Supplementary Table S6).

### 3.3 Association between vitamin D level and OAB symptom severity

We further explored the relationship between serum 25(OH)D concentrations and OAB symptom severity based on OABSS categories. A consistent inverse association was observed, indicating that higher 25(OH)D concentrations were associated with lower odds of experiencing moderate or severe OAB symptoms (Table 3). Compared with female with serum 25(OH)D concentrations <25 nmol/L, those with concentrations of >75 nmol/L nmol/L had significantly lower odds of moderate (OR: 0.540, 95% CI: 0.412–0.709, *p* < 0.001) and severe (OR: 0.607, 95% CI: 0.435–0.849, *p* = 0.004) OAB symptoms. Similar protective associations were observed in the intermediate 25(OH)D categories (25–49.9 and 50–74.9 nmol/L), with a clear trend of decreasing symptom severity as vitamin D levels increased. In contrast, no statistically significant associations were observed between 25(OH)D levels and OAB symptom severity among male participants (Table 3). These findings suggest a potential sex-specific relationship, in which higher 25(OH)D concentrations are associated with reduced symptom burden among female with OAB.

**Table 3:** Association between serum 25(OH)D concentration and OABSS.

Variables (%)	Fully adjusted model		
	Mild OABSS = 3	Moderate OABSS = 4	Severe OABSS = 5, 6
Total			
Log_10_(25(OH)D)	0.899 (0.799,1.012) 0.077	0.720 (0.594, 0.872) < 0.001	0.708 (0.545, 0.920) < 0.001
<25	1.0	1.0	1.0
25–49.9	0.910 (0.759,1.091) 0.309	0.720 (0.593, 0.875) < 0.001	0.799 (0.690, 0.925) 0.003
50–74.9	0.895 (0.745,1.074) 0.236	0.670 (0.555, 0.809) < 0.001	0.778 (0.684, 0.886) < 0.001
>75	0.865 (0.715,1.047) 0.137	0.624 (0.509, 0.765) < 0.001	0.706 (0.611, 0.815) < 0.001
Female			
Log_10_(25(OH)D)	0.882 (0.751,1.035)0.124	0.622 (0.486, 0.796) < 0.001	0.609 (0.450, 0.824) < 0.001
<25	1.0	1.0	1.0
25–49.9	0.938 (0.741,1.189)0.599	0.651 (0.503, 0.842) < 0.001	0.718 (0.524, 0.986)0.040
50–74.9	0.873 (0.687,1.111)0.266	0.612 (0.475, 0.788) < 0.001	0.680 (0.497, 0.931)0.016
>75	0.866 (0.674,1.112)0.260	0.540(0.412, 0.709) < 0.001	0.607 (0.435, 0.849)0.004
Male			
Log_10_(25(OH)D)	0.993 (0.836,1.181)0.940	0.964 (0.712, 1.306)0.814	1.255 (0.883, 1.784)0.206
<25	1.0	1.0	1.0
25–49.9	0.875 (0.652,1.174)0.372	0.865 (0.639, 1.170)0.346	1.204 (0.926, 1.565)0.166
50–74.9	0.913 (0.680,1.226)0.546	0.783 (0.587, 1.046)0.098	1.237 (0.994, 1.54)0.056
>75	0.882 (0.648,1.201)0.425	0.809 (0.593, 1.103)0.180	1.237 (0.961, 1.592)0.098

Fully adjusted model adjusts for age, race, marital status, poverty income ratio, education level, smoking, drinking, hypertension, diabetes, BMI, asthma, COPD, CVD, SII, vitamin D supplement use, season of blood collection and physical activity MET were adjusted.

### 3.4 Association between vitamin D supplementation and OAB

To further examine the relationship between vitamin D supplementation and OAB, we constructed weighted logistic regression models (Supplementary Table S7). In the unadjusted model (Model 1), vitamin D supplementation was significantly associated with an increased risk of OAB in the total population (OR: 1.265, 95% CI: 1.187–1.348, *p* < 0.001), in females (OR: 1.092, 95% CI: 1.004–1.187, *p* = 0.039), and in males (OR: 1.413, 95% CI: 1.280–1.560, *p* < 0.001). After adjusting for sociodemographic factors (Model 2), the association reversed, with vitamin D supplementation showing a protective effect in the total population (OR: 0.928, 95% CI: 0.863–0.998, *p* = 0.044) and in females (OR: 0.874, 95% CI: 0.795–0.961, *p* = 0.006), while no significant association was observed in males (OR: 1.031, 95% CI: 0.921–1.154, *p* = 0.597). In the fully adjusted model (Model 3), the association remained marginally non-significant in the total population (OR: 0.932, 95% CI: 0.865–1.003, *p* = 0.061) but was still significant in females (OR: 0.894, 95% CI: 0.811, 0.984, *p* = 0.023). No significant association was found in males (OR: 1.007, 95% CI: 0.898–1.129, *p* = 0.910; Supplementary Table S7).

### 3.5 Nonlinear correlation and subgroup analyses between 25(OH)D concentrations and OAB

**Figure 2:**
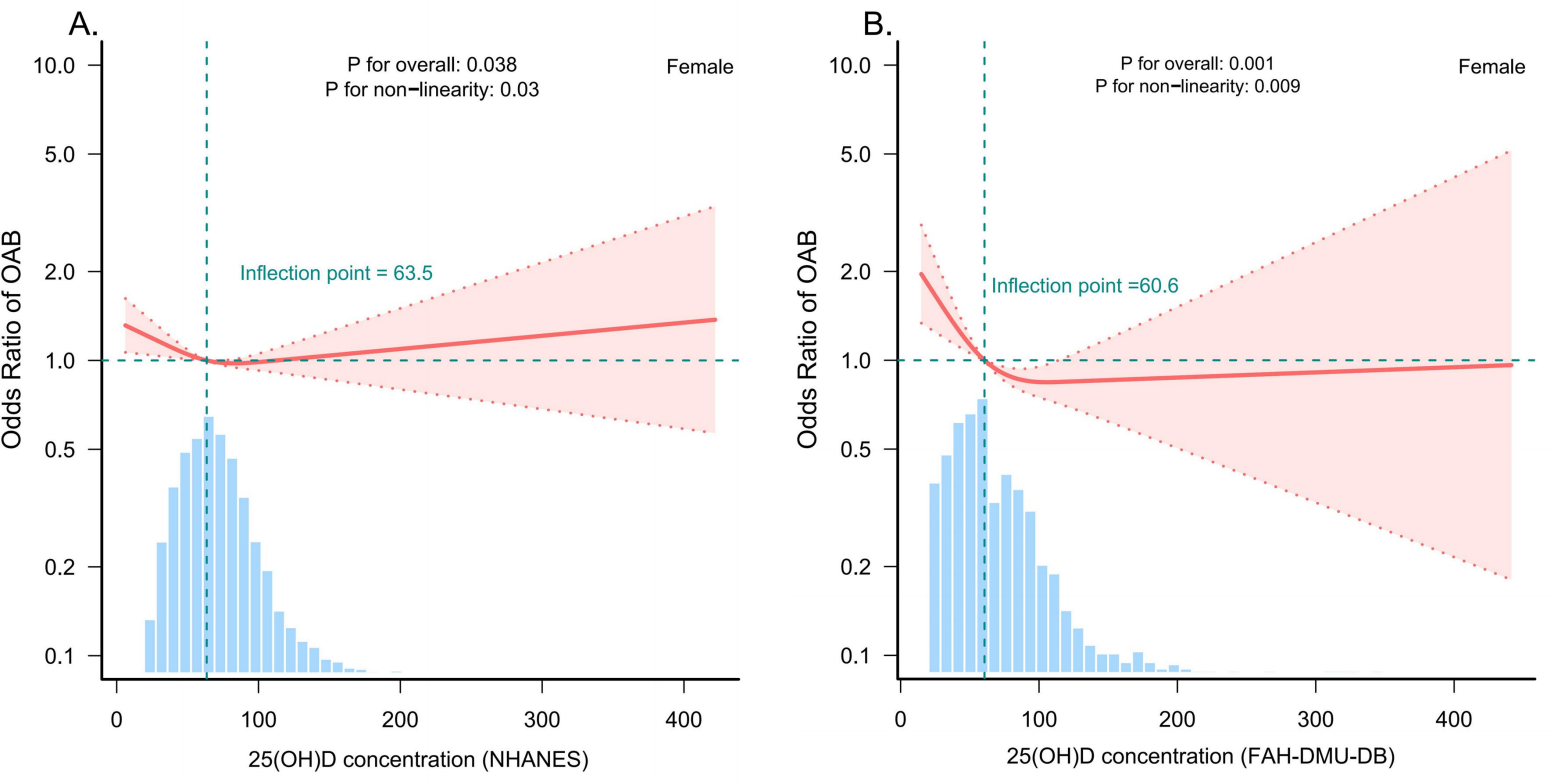
Dose–response association between serum 25(OH)D concentrations and the risk of OAB in females. The solid line represents the estimated odds ratio (OR), and the shaded area indicates the 95% confidence interval (CI), derived from restricted cubic spline (RCS) regression.

Among female, a non-linear association between serum 25(OH)D concentrations and the risk of OAB was identified using smoothing curve analyses. In the NHANES cohort, a statistically significant overall relationship was observed (*p* = 0.038), with clear evidence of nonlinearity in female (P for nonlinearity = 0.030; Figure 2A; Supplementary Figure S4). Subsequent threshold effect analysis revealed an inflection point at 63.5 nmol/L, indicating a change in the pattern of association: 25(OH)D levels below this threshold were more strongly associated with a reduction in OAB risk, while levels above this point showed a plateau effect (Supplementary Table S8). In contrast, no significant nonlinear relationship was observed in male participants, suggesting sex-specific differences in the dose–response pattern between 25(OH)D status and OAB risk.

Consistent findings were observed in the FAH-DMU-DB cohort, with a significant overall relationship (*p* = 0.001) and nonlinearity (P for nonlinearity = 0.009) between serum 25(OH)D concentrations and the risk of OAB (Figure 2B). The inflection point in this cohort was 60.6 nmol/L, suggesting a similar pattern in which increases in 25(OH)D below this threshold were associated with a greater reduction in OAB risk, while higher levels demonstrated minimal additional benefit.

To further investigate the potential nonlinear association between the variable of interest and the outcome, we employed a two-piecewise logistic regression model (Supplementary Table S9). In the fully adjusted standard logistic regression model, a statistically significant inverse association was observed (OR: 0.998 [95% CI, 0.997–1.000], *p* = 0.044). Below this threshold (<63.5), the variable was significantly associated with decreased odds of the outcome (OR: 0.995 [95% CI, 0.991–0.999], *p* = 0.009). However, above this threshold (≥63.5), no significant association was observed (OR: 1.000 [95% CI, 0.998–1.002], *p* = 0.992; Supplementary Table S9).

**Figure 3:**
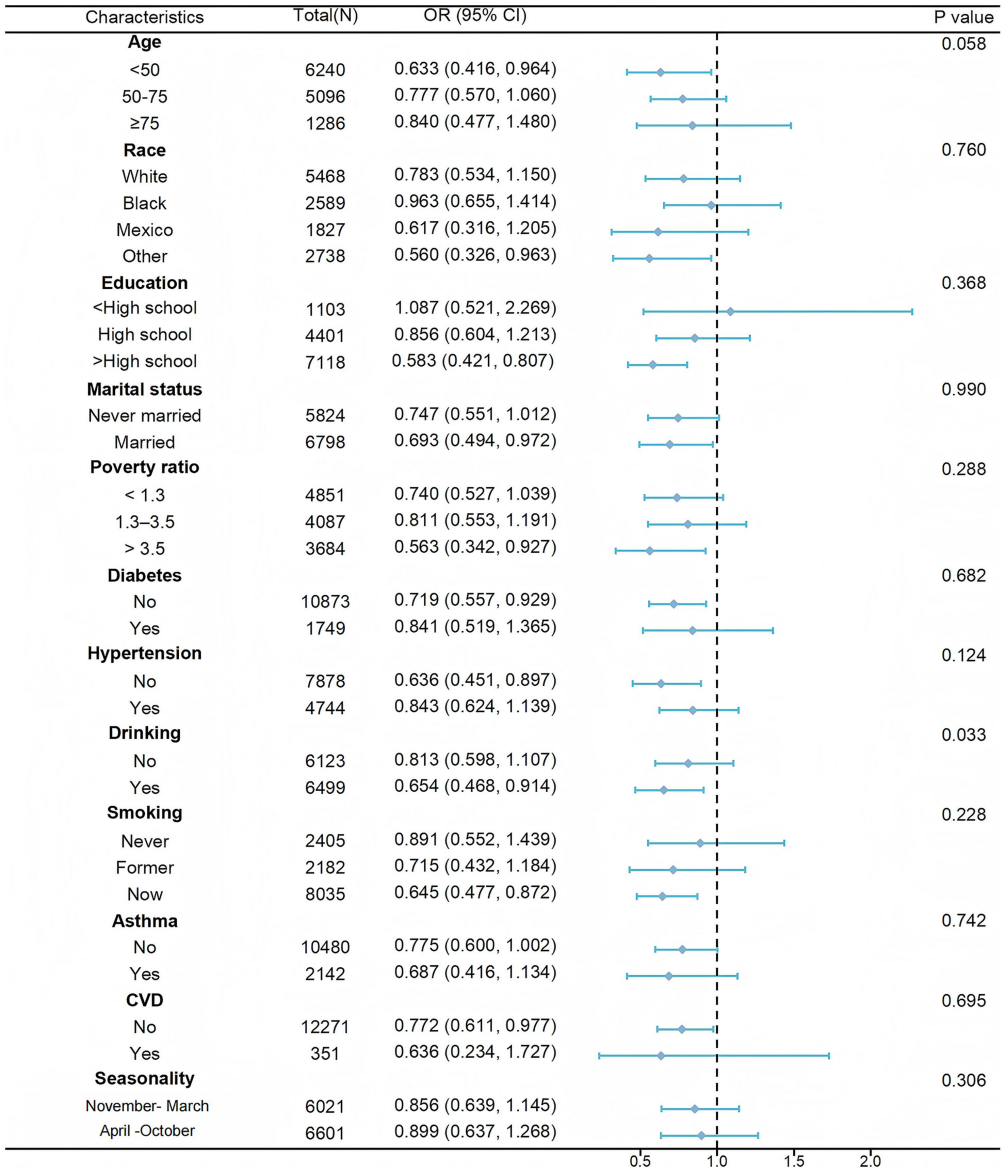
Subgroup analysis of the association of the serum 25(OH)D concentration and OAB in female. Analyses were adjusted for age, race, marital status, poverty income ratio, education level, smoking, drinking, hypertension, diabetes, BMI, asthma, COPD, CVD, SII, season of blood collection, vitamin D supplement use, and physical activity MET were adjusted.

To further explore potential effect modifiers in the relationship between 25(OH)D concentrations and OAB, stratified and interaction analyses were conducted (Figure 3). Subgroup analyses were performed according to age (<50, 50–75, and ≥75 years old), race (White, Black, Mexico and other race), education level (below high school, high school, and above high school), PIR (≤1.3, 1.3–3.5, and >3.5), marital status (married versus nerver married), presence of diabetes or hypertension (yes / no), and smoking statues (never, former, current). Across these subgroups, the association between 25(OH)D concentrations and OAB prevalence remained generally consistent, with no significant effect modification observed. However, a significant interaction was observed with alcohol consumption (P for interaction = 0.033). Among female who reported alcohol intake, higher vitamin D levels were associated with a greater reduction in OAB risk (OR: 0.654, 95% CI: 0.468–0.914), compared to those who did not consume alcohol (OR: 0.813, 95% CI: 0.598–1.107; Figure 3). These findings suggest that alcohol consumption may modify the protective effect of 25(OH)D on OAB prevalence.

### 3.6 Mediation and suppression effects of inflammatory biomarkers on the association between 25(OH)D and OAB prevalence

To explore the role of systemic inflammation in the association between serum 25(OH)D levels and OAB, we examined the relationships between inflammatory biomarkers and OAB and assessed their potential moderating or suppression effects. In the NHANES cohort (Supplementary Table S10), fully adjusted models showed that SII (OR = 1.001, 95% CI: 1.0001–1.0004, *p* = 0.001), neutrophil count (OR = 1.068, 95% CI: 1.0397–1.0960, *p* < 0.001), and NLR (OR = 1.054, 95% CI: 1.014–1.096, *p* = 0.008) were positively associated with OAB, whereas WBC was not significant (OR = 1.009, 95% CI: 0.9925–1.0254, *p* = 0.291). In the FAH-DMU-DB cohort (Supplementary Table S11), SII (OR = 1.0003, 95% CI: 1.0001–1.0006, *p* < 0.001) and NLR (OR = 1.171, 95% CI: 1.104–1.241, *p* < 0.001) were positively associated with OAB, while neutrophil count showed an inverse association (OR = 0.905, 95% CI: 0.861–0.951, *p* < 0.001) and WBC was not significant (OR = 0.964, 95% CI: 0.921–1.009, *p* = 0.116).

**Figure 4:**
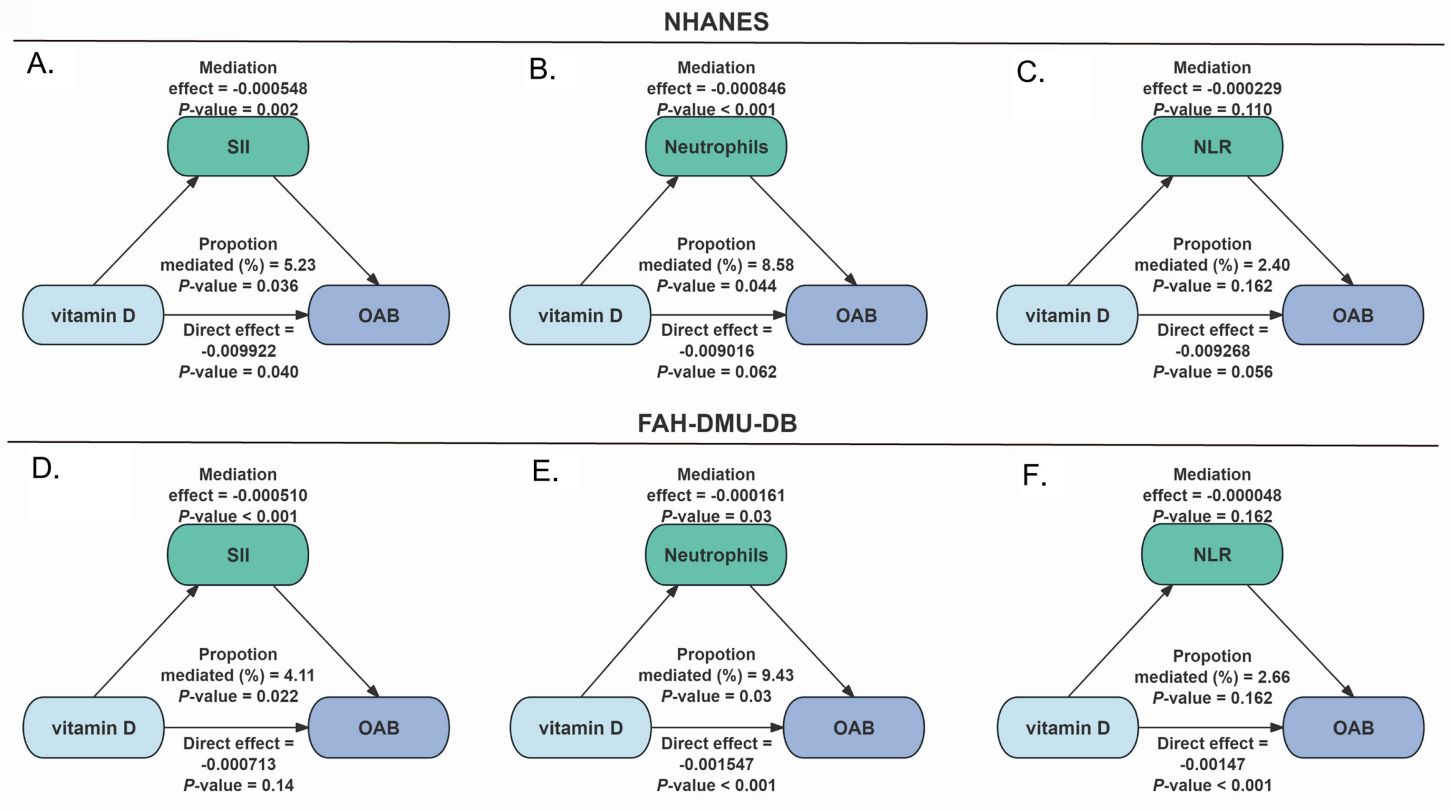
Path diagrams illustrating the mediation effects of inflammatory biomarkers on the association between serum 25(OH)D concentrations and OAB in females. **(A–C)** Depict models testing the mediating roles of **(A)** SII, **(B)** neutrophil count, and **(C)** NLR in NHANES. **(D,E)** Depict models testing the mediating roles of **(A)** SII, **(B)** neutrophil count, and **(C)** NLR in FAH-DMU-DB. All mediation models were adjusted for age, race, marital status, poverty income ratio, education level, smoking, drinking, hypertension, diabetes, BMI, asthma, COPD, CVD, SII, season of blood collection, vitamin D supplement use, and physical activity MET were adjusted.

Mediation analyses were conducted to assess whether systemic inflammation may statistically account for part of the inverse association between serum 25(OH)D levels and OAB. In the NHANES cohort, inclusion of SII and neutrophil count reduced the magnitude of the association between 25(OH)D and OAB by 5.23 and 8.58%, respectively (both *p* < 0.05; Figure 4A). Similar attenuation patterns were observed in the FAH-DMU-DB cohort, with reductions of 4.11 and 9.43% after adjustment for SII and neutrophil count, respectively (both p < 0.05; Figure 4B). In contrast, NLR did not materially influence the association between 25(OH)D and OAB in either cohort. Notably, the directions of the effects of 25(OH)D and inflammatory biomarkers on OAB were opposite, which may suggest a potential role of systemic inflammation in the observed association.

### 3.7 MR analysis

We conducted a two-sample MR analysis to investigate the potential association between vitamin D levels and urinary incontinence/ bladder problem. The IVW method identified a significant potential effect, indicating higher vitamin D levels are associated with a reduced risk of “Unspecified urinary incontinence” (OR = 0.998, 95% CI: 0.997–0.999, *p* = 0.008) and “Bladder problem” (OR = 0.998, 95% CI: 0.996–1.000, *p* = 0.026), with the MR-Egger analysis showing the significance for urinary incontinence (OR = 0.997, *p* = 0.008; Supplementary Figure S5). Furthermore, sensitivity analyses indicated no evidence of heterogeneity across instrumental variables (Cochran’s Q *p* > 0.05), nor evidence of directional horizontal pleiotropy as assessed by the MR-Egger intercept test (*p* > 0.05; Supplementary Table S11). In addition, the MR-PRESSO analysis did not identify any outlier variants, further supporting the stability of the IVW estimate (Supplementary Table S12).

## 4 Discussion

In this study, we investigated the association between serum 25(OH)D levels and the prevalence of OAB in male and female. Our findings demonstrated that higher serum 25(OH)D concentrations were significantly associated with a reduced risk of OAB in female, whereas no significant association was observed in male. Furthermore, when OAB severity was categorized into mild, moderate, and severe based on the OABSS, elevated 25(OH)D levels were significantly associated with a lower risk of developing moderate or severe symptoms in female. Notably, our mediation and suppression effects analysis showed that inflammatory biomarkers attenuated the protective association between 25(OH)D and OAB. MR analysis further supported a potential association between vitamin D level and urinary incontinence/bladder problem.

Previous studies investigating the relationship between vitamin D and OAB have yielded inconsistent findings. While some reported no significant association between serum vitamin D levels and OAB, others suggested that vitamin D deficiency may worsen OAB symptoms, particularly among men during the winter months (4, 10). In our analysis, we identified a significant association between serum 25(OH)D levels and OAB in the overall population. Several factors may explain these discrepancies across studies. Many prior investigations were limited by sex-specific imbalances in sample size, particularly smaller male cohorts. Additionally, differences in vitamin D quantification methods could contribute to heterogeneity. Earlier studies used radioimmunoassay, which may produce less precise or systematically biased estimates, whereas others employed LC–MS/MS, the current gold standard for 25(OH)D measurement due to its superior specificity and accuracy.

In sex-stratified analyses, we observed a clear inverse association between higher serum vitamin D levels and OAB risk in females, but no corresponding association in males. Several mechanisms may account for the stronger association observed in women. First, vitamin D may improve lower urinary tract function in females by enhancing pelvic floor muscle strength through its effects on skeletal muscle, increasing urethral sphincter tone, and inhibiting detrusor muscle overactivity (24, 25). Vitamin D receptors are expressed in both the urothelium and detrusor muscle, with higher expression reported in female bladder tissues, potentially increasing responsiveness to circulating vitamin D. In addition, estrogen has been shown to upregulate VDR expression and potentiate vitamin D–mediated anti-inflammatory effects, which may be particularly relevant in postmenopausal women who are often deficient in both estrogen and vitamin D (26, 27). Furthermore, females may exhibit more robust innate immune responses and heightened sensitivity to inflammatory signaling, thereby amplifying the physiological influence of vitamin D status on bladder function (28).

Non-linear analysis using smoothing curves revealed a significant association between serum vitamin D levels and the risk of OAB. A statistically significant overall relationship was observed (*p* = 0.008), with clear evidence of nonlinearity in woman. As vitamin D levels increased toward approximately 63.5 nmol/L, the risk of OAB decreased markedly. Beyond this inflection point, the curve began to flatten, suggesting a plateau in the protective effect. Notably, this threshold lies below the commonly cited sufficiency cutoff for bone health (≥75 nmol/L), implying that the optimal vitamin D level for lower urinary tract function may differ from that for skeletal outcomes. This finding raises the possibility of bladder-specific mechanisms, such as vitamin D–mediated modulation of detrusor muscle contractility or urethral sphincter tone, which merit further mechanistic investigation. The steep decline in risk prior to the 63.5 nmol/L threshold also underscores the importance of correcting vitamin D deficiency as a potential strategy to mitigate OAB symptoms.

Our mediation analysis further revealed that both the SII and neutrophil count partly mediated the negative association between serum 25(OH)D and OAB. Pathological studies have demonstrated increased infiltration and activation of systemic inflammatory cells, including mast cells, T lymphocytes, and B lymphocytes within the detrusor muscle of individuals with OAB (29). The subsequent release of cytokines and other pro-inflammatory mediators initiates a localized inflammatory response that contributes to OAB (29–31). Notably, Vitamin D’s well-established antibacterial (32, 33), anti-inflammatory (34), and antiproliferative properties likely underlie this effect by reducing pro-inflammatory cell activity and cytokine release within bladder tissue (35, 36). Indeed, experimental studies directly support vitamin D’s anti-inflammatory effects in bladder tissue. For instance, human bladder smooth muscle cells have been shown to exhibit reduced NF-κB activation and lower expression of pro-inflammatory cytokines (IL-6, TNF-*α*, IL-1β) following treatment with calcitriol (active vitamin D) (37). Moreover, vitamin D has been demonstrated to suppress mast cell activation and degranulation, including IgE-mediated histamine release, via mast cell expression of vitamin D receptor and conversion of 25(OH)D to active 1,25(OH)₂D₃ through CYP27B1 enzymatic activity (38). In our study, mediation analysis revealed SII, a composite marker of systemic inflammation, mediated the negative association between serum 25(OH)D and OAB. Considering the mechanistic evidence, it seems plausible that higher vitamin D levels blunt systemic inflammatory signaling, leading to lower SII, which in turn may reduce bladder inflammatory burden and OAB symptoms. Therefore, these inflammatory biomarkers thus provided valuable insight into the potential biological mechanisms linking vitamin D and OAB.

Our study found that SII, a composite marker of systemic inflammation, significantly mediated the negative association between serum 25(OH)D and OAB in the NHANES cohort. This suggests that higher vitamin D levels may blunt systemic inflammatory signaling, thereby reducing the bladder inflammatory burden. However, regarding neutrophil counts, we observed substantial heterogeneity between the two study populations. In NHANES, higher neutrophil counts were associated with increased OAB risk, consistent with the classic role of chronic inflammation in bladder dysfunction. Conversely, in the FAH-DMU-DB clinical cohort, neutrophil counts showed an inverse association. This discrepancy likely stems from fundamental differences in population characteristics. NHANES represents a general population where elevated inflammatory markers typically reflect metabolic dysregulation. In contrast, the FAH-DMU-DB cohort is a hospital-based sample of patients presenting with urological symptoms, who generally have a higher burden of disease and older age. In such a high-risk clinical setting, immune dynamics can be complex; for instance, lower neutrophil counts in elderly clinical patients might reflect weakened immune response rather than low inflammation, potentially making them more susceptible to infections and OAB symptoms. Therefore, these findings suggest that while the vitamin D-inflammation-OAB pathway is evident in the general population, the specific role of immune cells may be modulated by the clinical status and severity of the disease.

This study also identified several factors potentially correlated with vitamin D status. In particular, we observed an interaction between alcohol intake and 25(OH)D level. Evidence from animal models suggests that chronic alcohol consumption can impair renal synthesis and/or accelerate the degradation of 1,25-dihydroxyvitamin D [1,25(OH)₂D], resulting in reduced circulating levels of the active form (39). Additionally, alcohol-induced liver dysfunction may disrupt hepatic 25-hydroxylation of vitamin D, while ethanol-related injury to the intestinal mucosa can impair vitamin D absorption (40). These mechanisms may collectively contribute to the interaction observed in our analysis. However, the subgroup and interaction analyses in this study are exploratory and hypothesis-generating rather than confirmatory. Although a statistically significant interaction with alcohol consumption was observed, this finding requires independent validation in future studies.

The present study possesses several notable strengths. First and foremost, our investigation is underpinned by a robust dual-cohort design that integrates the large-scale, nationally representative NHANES database with the FAH-DMU-DB clinical validation cohort, ensuring both the generalizability of findings to the US public and their replication in a specific high-risk clinical population. Second, to overcome the inherent limitations of observational designs, we incorporated a MR analysis. By utilizing genetic variants as instrumental variables, our MR analysis circumvents residual confounding, our MR analysis reduces the impact of residual confounding, providing genetically informed support for an association between vitamin D deficiency and OAB. Although exposure and outcome GWAS were derived from the same dataset, all instrumental variables were strong and multiple sensitivity analyses yielded consistent results, suggesting that potential bias due to sample overlap is unlikely to materially affect our conclusions. Third, this study offers novel mechanistic insights through mediation analysis, which explored the role of systemic inflammatory biomarkers (such as SII) in the pathway linking vitamin D deficiency to OAB, offering epidemiological evidence for the potential involvement of immune-inflammatory mechanisms.

Nevertheless, this study has certain limitations. First, despite the supportive evidence from our MR analysis, the primary observational component of both cohorts is cross-sectional. Therefore, while MR strengthens the case for causality, the observational results alone preclude definitive causal inferences and determination of temporal sequences. Second, the diagnosis of OAB was based on the OABSS questionnaire and self-reported symptoms rather than urodynamic testing, which remains the diagnostic gold standard. Although this is a widely accepted approach in large-scale epidemiological studies, it may introduce recall bias or potential misclassification. Besides, there is a difference in the definition of OAB across cohorts, with OABSS used in NHANES and ICD-10 diagnoses in the FAH-DMU-DB cohort. The NHANES definition is based on self-reported symptoms without clinical evaluation or exclusion of other potential causes, whereas the FAH-DMU-DB definition reflects physician-assigned diagnoses following routine clinical assessment. These differences in measurement approaches may introduce misclassification and affect comparability. However, in a sensitivity analysis using a more stringent OAB definition (OABSS ≥ 4), the association between serum 25(OH)D levels and OAB remained consistent, supporting the robustness of our findings despite differences in measurement approaches across the two cohorts. Future studies using consistent diagnostic criteria are needed to further confirm these results. Third, despite adjustment for a wide range of potential confounders, residual confounding from unmeasured factors—such as seasonality, sunlight exposure, and specific dietary calcium intake—cannot be completely excluded. Fourth, it is important to acknowledge that the FAH-DMU-DB cohort is hospital-based, which may introduce selection bias compared to the general population-based NHANES sample. Fifth, owing to the large number of subgroup analyses performed without correction for multiple testing, the risk of chance findings cannot be excluded. Specifically, the observed interaction between alcohol consumption and vitamin D levels should be interpreted with caution. We emphasize that these subgroup findings are exploratory and require verification in future studies to rule out false-positive associations. Last, both exposure and outcome GWAS were derived from the UK Biobank, which may introduce sample overlap and potentially violate the strict independence assumption of classical two-sample MR.

In summary, our findings highlight a significant inverse association between serum vitamin D levels and the risk of OAB in female. These results suggest that maintaining sufficient vitamin D levels may serve as an effective strategy for reducing the prevalence and severity of OAB, particularly among female populations. Nonetheless, given the observational design of this study, further longitudinal and interventional research is warranted to validate these associations and elucidate the underlying biological mechanisms.

## Data Availability

The datasets presented in this study can be found in online repositories. The names of the repository/repositories and accession number(s) can be found at: https://wwwn.cdc.gov/nchs/nhanes/Default.aspx.
