# Supplementary material for: Association between serum 25-hydroxyvitamin D concentrations and overactive bladder in female: a mediation analysis of inflammatory biomarkers
Source: Front Nutr. 2026 Jun 9;13:1741019. doi: 10.3389/fnut.2026.1741019 (PMC13286948; doi:10.3389/fnut.2026.1741019)
Supplement: Supplementary file 1 [file Data_Sheet_1.docx]

**Title: Association between serum 25-hydroxyvitamin D concentrations and overactive bladder: A mediation analysis of inflammatory biomarkers**


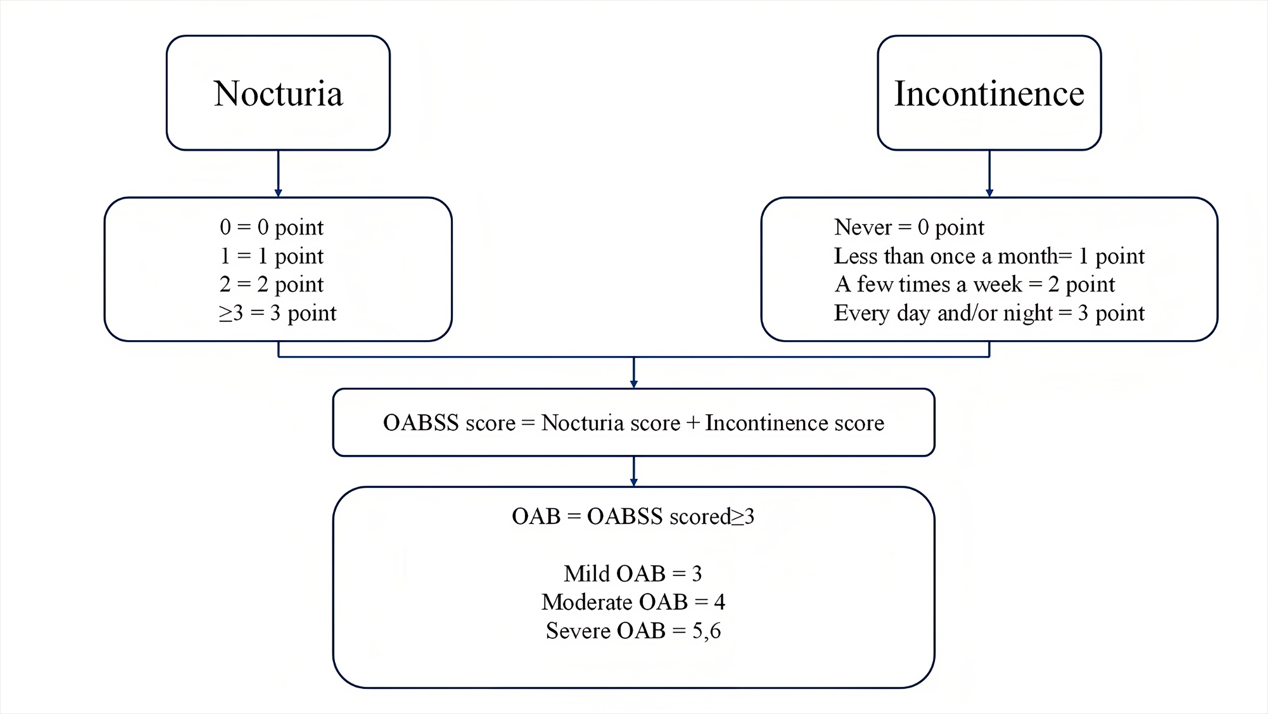


**Supplementary Figure S1. Flowchart of the OAB evaluation by utilizing OBASS questionnaire.**


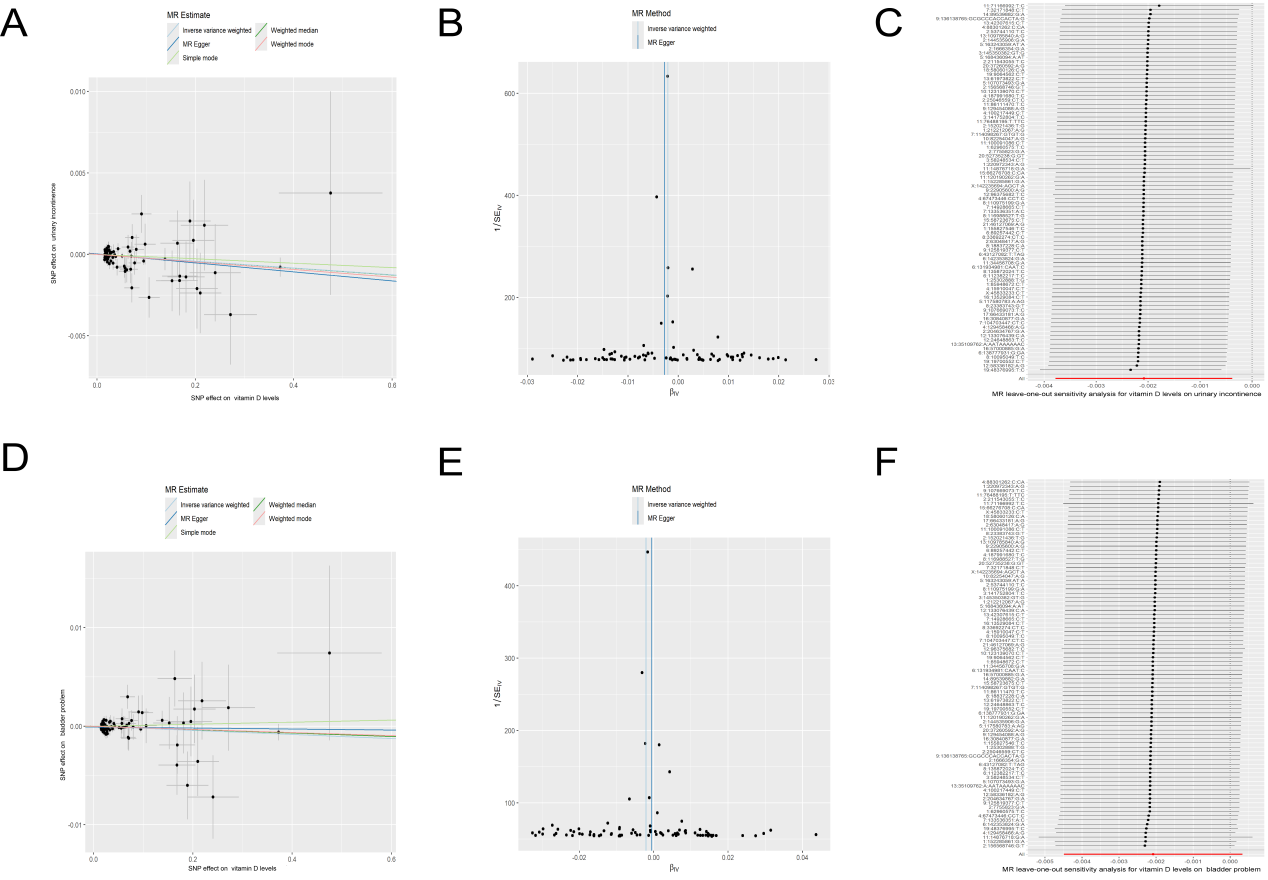


**Supplementary Figure S2. Mendelian randomization diagnostic plots.**

Results for Unspecified urinary incontinence (A–C) and Bladder problem (D–F). (A, D) Scatter plots depicting the causal effect estimates of individual genetic variants (SNPs). (B, E) Funnel plots assessing potential directional pleiotropy. (C, F) Leave-one-out sensitivity analyses to evaluate whether the causal association is driven disproportionately by any single SNP.


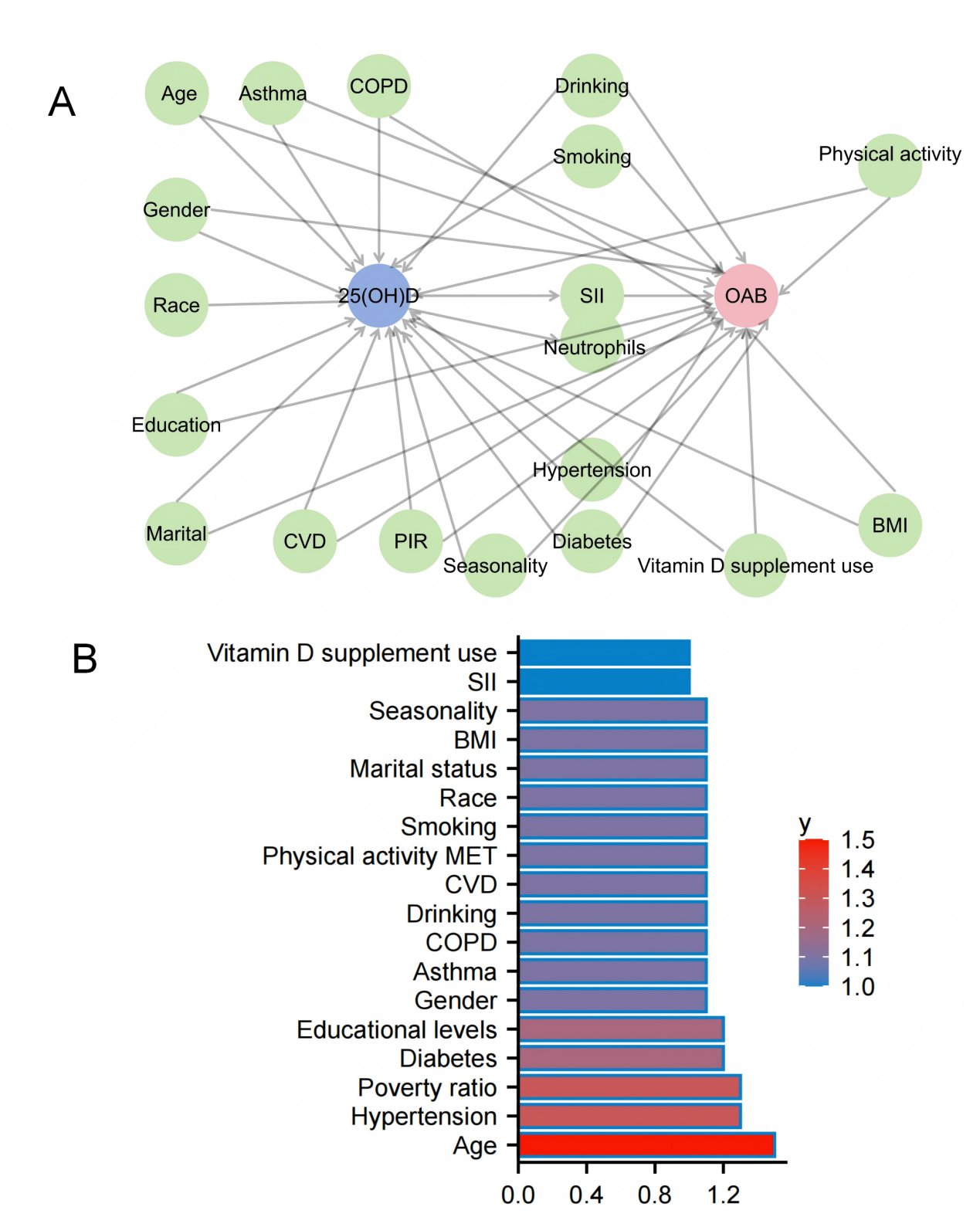


**Supplementary Figure S3.** Directed acyclic graph (DAG) framework to pre-identify variables with plausible causal relationships to both serum 25(OH)D levels and OAB. (A-B). Variance inflation factor (VIF) was analysed to assess multicollinearity among candidate covariates.

**
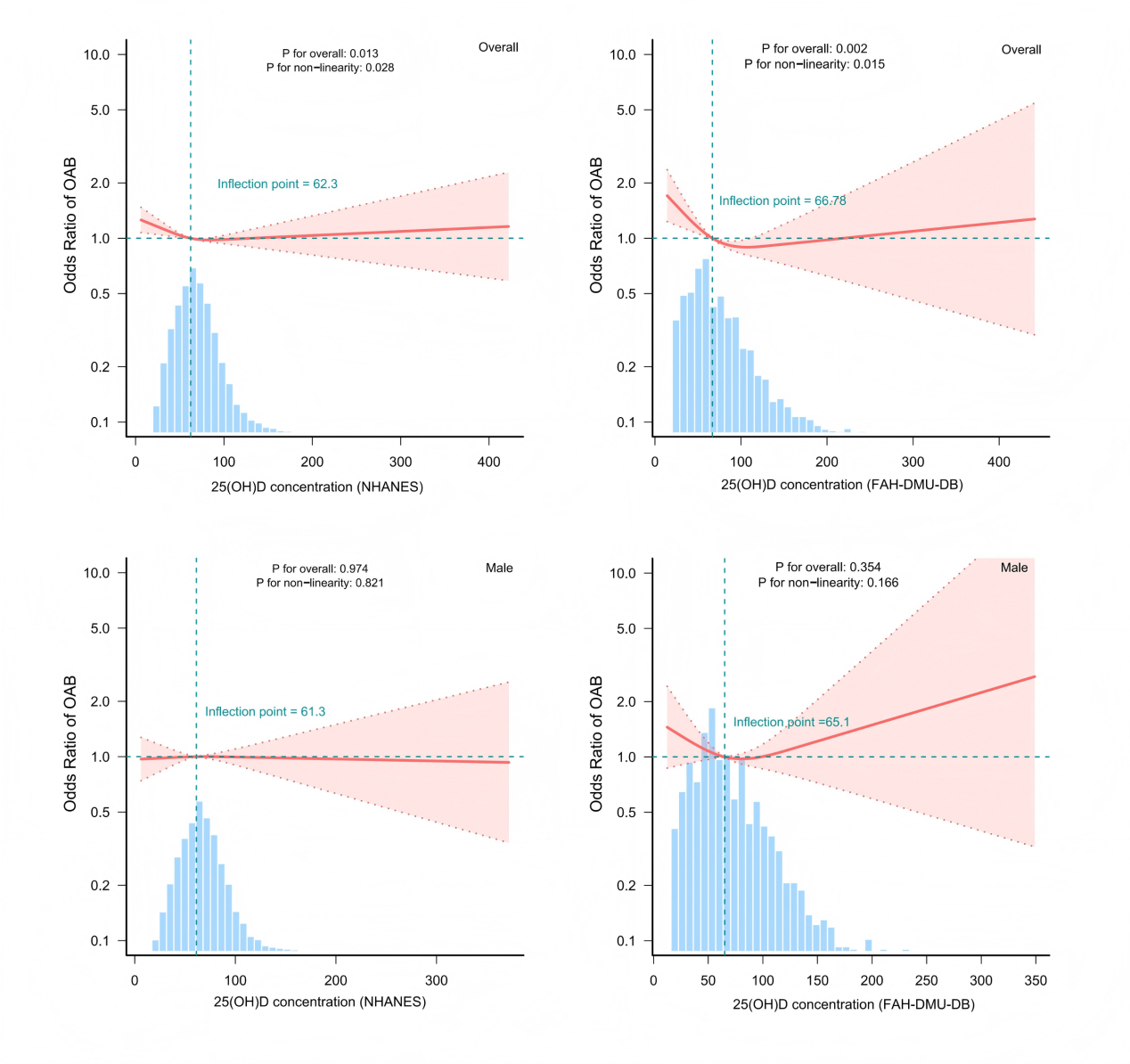
Supplementary Figure S4. Dose-response association between serum 25(OH)D concentrations and the risk of OAB**.

The solid line represents the estimated odds ratio (OR), and the shaded area indicates the 95% confidence interval (CI), derived from restricted cubic spline (RCS) regression.


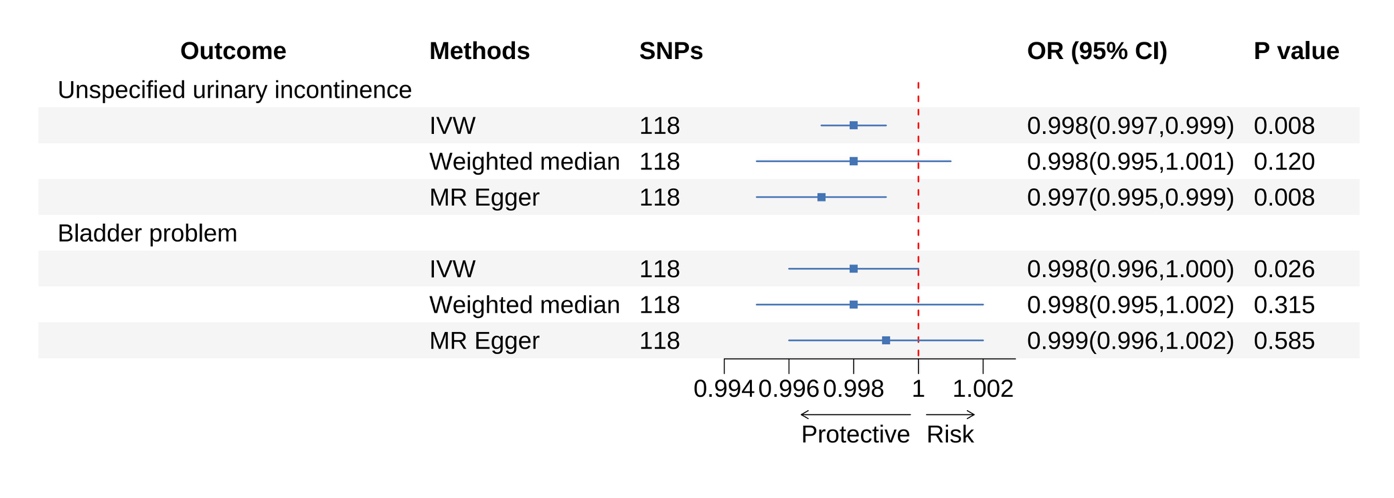
**Supplementary Figure S5. Two-sample MR was conducted to analysis the causal association between vitamin D levels and urinary incontinence/ bladder problem.**

The IVW method showed that higher Vitamin D levels are associated with a reduced risk of “Unspecified urinary incontinence” (OR = 0.998, 95% CI: 0.997–0.999, P = 0.008) and “Bladder problem” (OR = 0.998, 95% CI: 0.996–1.000, P = 0.026)


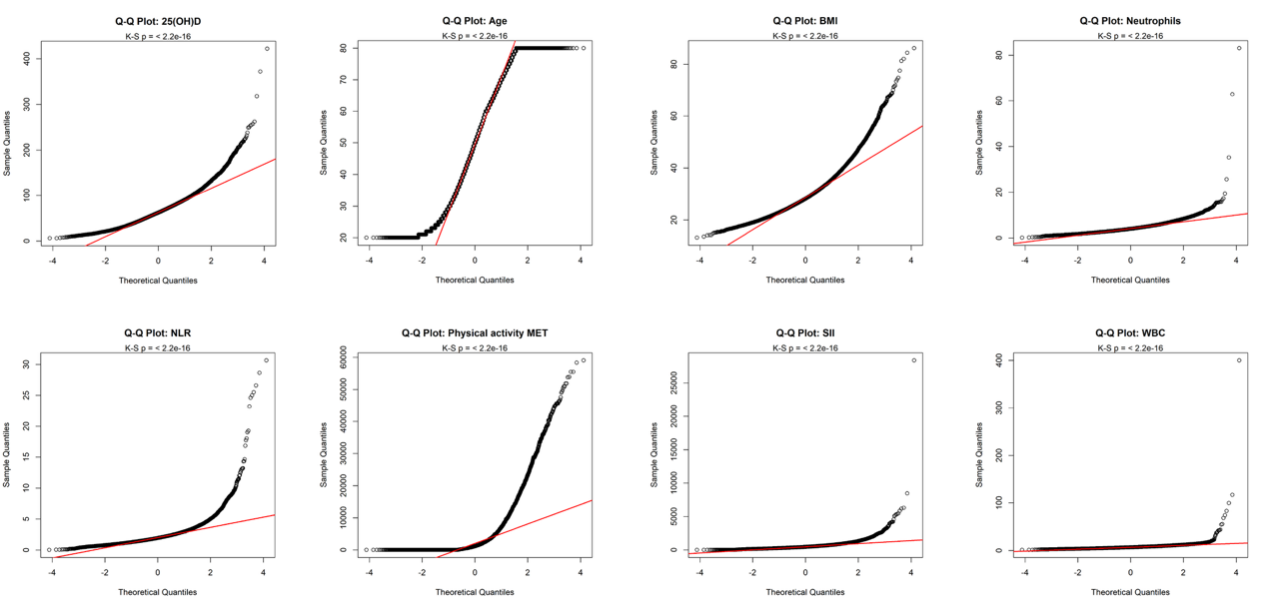


**Supplementary Figure S6. Q-Q chart**

**Supplementary Table S1. Characteristics of the exposure and outcome GWAS summary statistics.**

| Items | UKB Phenotype Code | Gender | Year | Population |
| --- | --- | --- | --- | --- |
| Vitamin D | 30890_irnt | Female | 2018 | Europeans |
| urinary incontinence | R32 | Female | 2018 | Europeans |
| bladder problem | 20002_1201 | Female | 2017 | Europeans |

Note: The exposure and outcome datasets were obtained from the UK Biobank (Neale Lab).

**Supplementary Table S2. Features of 118 SNPs used as instrumental variables for exposure (vitamin D levels).**

| **SNP** | **EA** | **OA** | **EAF** | **effect** | **SE** | **N** | **P-value** | **F.Statistic** |
| --- | --- | --- | --- | --- | --- | --- | --- | --- |
| rs61816761 | A | G | 0.023268 | 0.13816 | 0.011834 | 194,174 | 1.77E-31 | 168.613633 |
| rs7556519 | C | T | 0.29095 | 0.018714 | 0.0037089 | 194,174 | 4.52E-07 | 28.06130289 |
| rs3750296 | C | G | 0.34168 | -0.020279 | 0.0035503 | 194,174 | 1.12E-08 | 35.92912421 |
| rs138741141 | G | A | 0.0023954 | 0.1684 | 0.036124 | 194,174 | 3.14E-06 | 26.32062079 |
| rs867772 | G | A | 0.68453 | 0.018983 | 0.0036313 | 194,174 | 1.72E-07 | 30.22487018 |
| rs6698680 | G | A | 0.46526 | -0.015834 | 0.0033819 | 194,174 | 2.84E-06 | 24.22648747 |
| rs1048712786 | GT | G | 0.497 | -0.016606 | 0.0034106 | 194,174 | 1.12E-06 | 26.77508899 |
| rs181207267 | G | T | 0.0013989 | -0.21847 | 0.048321 | 194,174 | 6.15E-06 | 25.89624792 |
| rs7522116 | T | C | 0.56636 | 0.01543 | 0.0034158 | 194,174 | 6.27E-06 | 22.71021012 |
| rs1168040 | C | T | 0.66186 | 0.017704 | 0.0035923 | 194,174 | 8.31E-07 | 27.24476194 |
| rs7541151 | T | C | 0.017877 | -0.063095 | 0.012894 | 194,174 | 9.92E-07 | 27.14736773 |
| rs113327160 | T | C | 0.02247 | -0.055543 | 0.012174 | 194,174 | 5.06E-06 | 26.31887341 |
| rs224044 | A | G | 0.57044 | -0.017973 | 0.0034111 | 194,174 | 1.37E-07 | 30.74399573 |
| rs6586030 | G | A | 0.84512 | 0.023436 | 0.0046822 | 194,174 | 5.59E-07 | 27.92285023 |
| rs12789536 | T | C | 0.38088 | -0.018216 | 0.0034824 | 194,174 | 1.69E-07 | 30.39160296 |
| rs964184 | C | G | 0.86923 | -0.036215 | 0.004997 | 194,174 | 4.27E-13 | 57.91164721 |
| rs11217815 | A | G | 0.38949 | -0.016285 | 0.0034801 | 194,174 | 2.88E-06 | 24.49266245 |
| rs116970203 | A | G | 0.028051 | -0.37246 | 0.010184 | 194,174 | 1.00E-200 | 1480.01262 |
| rs117906197 | A | G | 0.011418 | 0.079788 | 0.015855 | 194,174 | 4.85E-07 | 27.90981224 |
| rs1313832805 | GT | G | 0.56565 | -0.017499 | 0.003603 | 194,174 | 1.19E-06 | 29.22106441 |
| rs1406474097 | G | T | 0.45702 | 0.015661 | 0.0033976 | 194,174 | 4.04E-06 | 23.63891215 |
| rs766406857 | C | T | 0.79026 | -0.094924 | 0.0041447 | 194,174 | 6.47E-116 | 581.7268346 |
| rs200974687 | TTC | T | 0.17022 | 0.021897 | 0.0045137 | 194,174 | 1.23E-06 | 26.30378304 |
| rs188866784 | C | T | 0.0031015 | 0.15243 | 0.032548 | 194,174 | 2.83E-06 | 27.90246875 |
| rs4764726 | G | C | 0.47123 | -0.015456 | 0.0034033 | 194,174 | 5.59E-06 | 23.11863889 |
| rs61952071 | A | C | 0.31351 | 0.019022 | 0.003641 | 194,174 | 1.75E-07 | 30.24699359 |
| rs16927657 | C | T | 0.014809 | -0.071517 | 0.014283 | 194,174 | 5.53E-07 | 28.98318878 |
| rs10744561 | G | C | 0.59227 | -0.016362 | 0.0034342 | 194,174 | 1.89E-06 | 25.10949255 |
| rs576081622 | G | A | 0.0069901 | -0.090564 | 0.02036 | 194,174 | 8.67E-06 | 22.11129665 |
| rs10859995 | C | T | 0.58404 | 0.051038 | 0.0034281 | 194,174 | 4.21E-50 | 246.0639927 |
| rs9514988 | G | A | 0.36599 | -0.015771 | 0.0035259 | 194,174 | 7.73E-06 | 22.415608 |
| rs1871199914 | AATAAAAAAC | A | 0.36924 | 0.016004 | 0.0035051 | 194,174 | 4.98E-06 | 23.16852515 |
| rs1157876727 | T | C | 0.098778 | -0.025145 | 0.0056667 | 194,174 | 9.11E-06 | 21.86053491 |
| rs150210012 | T | C | 0.019784 | -0.057041 | 0.012636 | 194,174 | 6.36E-06 | 24.50651526 |
| rs520469 | C | T | 0.51377 | -0.015455 | 0.0033789 | 194,174 | 4.79E-06 | 23.17485052 |
| rs8018720 | C | G | 0.82186 | 0.041458 | 0.0043986 | 194,174 | 4.34E-21 | 97.77125969 |
| rs143524217 | A | G | 0.0074056 | -0.10595 | 0.021481 | 194,174 | 8.13E-07 | 32.0495747 |
| rs7181542 | C | T | 0.48952 | 0.015892 | 0.0033786 | 194,174 | 2.56E-06 | 24.51194115 |
| rs1800588 | T | C | 0.21453 | -0.035843 | 0.0041059 | 194,174 | 2.58E-18 | 84.1067818 |
| 15:66276708:C:CA | CA | C | 0.0010801 | -0.24039 | 0.052187 | 194,174 | 4.10E-06 | 24.2157645 |
| rs34289026 | T | C | 0.21555 | -0.01862 | 0.0042028 | 194,174 | 9.41E-06 | 22.76880147 |
| rs7500719 | A | G | 0.27127 | -0.018774 | 0.0037924 | 194,174 | 7.42E-07 | 27.06195494 |
| rs12720922 | A | G | 0.18321 | 0.02009 | 0.004357 | 194,174 | 4.01E-06 | 23.45786722 |
| rs2091136687 | C | CA | 0.54568 | -0.017395 | 0.0035699 | 194,174 | 1.10E-06 | 29.1360397 |
| rs1907394458 | T | TAAA | 0.44608 | 0.015438 | 0.003467 | 194,174 | 8.47E-06 | 22.87228876 |
| rs1330907905 | G | A | 0.77346 | -0.020468 | 0.0040486 | 194,174 | 4.29E-07 | 28.51109753 |
| rs4121823 | A | T | 0.84151 | 0.023483 | 0.0046596 | 194,174 | 4.67E-07 | 28.56595777 |
| rs17773774 | A | C | 0.20009 | -0.019875 | 0.0042235 | 194,174 | 2.53E-06 | 24.55569913 |
| rs2061514926 | T | C | 0.08491 | 0.042697 | 0.0060503 | 194,174 | 1.71E-12 | 55.02461672 |
| rs212100 | C | T | 0.83566 | 0.067009 | 0.0045465 | 194,174 | 3.89E-49 | 239.768274 |
| rs1261000197 | A | AT | 0.57564 | -0.015924 | 0.0035551 | 194,174 | 7.50E-06 | 24.0580348 |
| rs117552735 | T | C | 0.017479 | -0.060322 | 0.013488 | 194,174 | 7.74E-06 | 24.27061435 |
| rs56326655 | A | G | 0.039202 | -0.040661 | 0.0087745 | 194,174 | 3.59E-06 | 24.18617091 |
| rs1681682117 | G | T | 0.0024413 | 0.16789 | 0.037242 | 194,174 | 6.55E-06 | 26.66155638 |
| rs137976364 | T | G | 0.013474 | 0.068417 | 0.014912 | 194,174 | 4.48E-06 | 24.16594482 |
| rs150094097 | G | C | 0.047343 | 0.038297 | 0.0080258 | 194,174 | 1.83E-06 | 25.69188128 |
| rs114311700 | A | G | 0.019195 | 0.057259 | 0.012773 | 194,174 | 7.38E-06 | 23.97333873 |
| rs72925104 | C | G | 0.28484 | -0.017758 | 0.0037623 | 194,174 | 2.36E-06 | 24.94966368 |
| rs231390 | A | G | 0.67579 | 0.017048 | 0.0036276 | 194,174 | 2.61E-06 | 24.7318699 |
| rs715 | C | T | 0.31125 | -0.018941 | 0.0036489 | 194,174 | 2.10E-07 | 29.87171706 |
| rs12475068 | G | C | 0.083269 | -0.046419 | 0.0061153 | 194,174 | 3.20E-14 | 63.89638925 |
| rs1252682906 | C | CT | 0.38315 | -0.015676 | 0.0034865 | 194,174 | 6.92E-06 | 22.55723987 |
| rs12712575 | A | T | 0.63823 | 0.01831 | 0.0035194 | 194,174 | 1.97E-07 | 30.06562896 |
| rs11887670 | C | T | 0.40111 | -0.015364 | 0.0034519 | 194,174 | 8.56E-06 | 22.02343228 |
| rs34503992 | G | A | 0.20194 | 0.019069 | 0.0042019 | 194,174 | 5.67E-06 | 22.76047058 |
| rs74348010 | A | G | 0.082119 | 0.032025 | 0.0061884 | 194,174 | 2.28E-07 | 30.02562528 |
| rs189778018 | G | A | 0.0011595 | 0.27138 | 0.053742 | 194,174 | 4.43E-07 | 33.12937667 |
| rs2092573355 | GT | G | 0.1879 | -0.038263 | 0.0044486 | 194,174 | 7.97E-18 | 86.79706038 |
| rs2838655 | G | A | 0.3433 | 0.016321 | 0.0036072 | 194,174 | 6.05E-06 | 23.32400433 |
| rs9848817 | C | T | 0.66804 | -0.015937 | 0.0035872 | 194,174 | 8.89E-06 | 21.8759486 |
| rs1163259274 | G | GT | 0.22304 | 0.022235 | 0.0041278 | 194,174 | 7.19E-08 | 33.27719467 |
| rs190126158 | G | C | 0.0036497 | 0.13362 | 0.029771 | 194,174 | 7.18E-06 | 25.21652018 |
| rs1347932730 | C | CT | 0.49354 | 0.018705 | 0.0034019 | 194,174 | 3.84E-08 | 33.96843206 |
| rs60106186 | T | C | 0.25597 | -0.017303 | 0.0038806 | 194,174 | 8.25E-06 | 22.14565143 |
| rs1972994 | T | A | 0.6504 | 0.01573 | 0.003541 | 194,174 | 8.91E-06 | 21.85117887 |
| rs150400074 | T | C | 0.018722 | 0.076612 | 0.012532 | 194,174 | 9.79E-10 | 41.88400207 |
| rs36029254 | G | A | 0.21277 | 0.020993 | 0.004126 | 194,174 | 3.62E-07 | 28.67092883 |
| rs56219219 | T | C | 0.12793 | 0.025873 | 0.0050525 | 194,174 | 3.05E-07 | 29.00673158 |
| rs549379886 | C | T | 0.0017156 | 0.20985 | 0.042773 | 194,174 | 9.30E-07 | 29.29344054 |
| rs78649910 | A | T | 0.10941 | -0.026289 | 0.0054532 | 194,174 | 1.43E-06 | 26.15520094 |
| rs747434642 | C | CCT | 0.098575 | -0.025358 | 0.0056659 | 194,174 | 7.62E-06 | 22.19180417 |
| rs11733890 | A | T | 0.34001 | -0.12895 | 0.0035708 | 194,174 | 1.00E-200 | 1459.961975 |
| rs1361997505 | CA | C | 0.13646 | -0.028015 | 0.0049786 | 194,174 | 1.84E-08 | 35.9224557 |
| rs2411044 | A | G | 0.20509 | -0.018871 | 0.0041989 | 194,174 | 6.99E-06 | 22.54858558 |
| rs1580553723 | AG | A | 0.38953 | -0.016607 | 0.0034881 | 194,174 | 1.93E-06 | 25.47189033 |
| rs1480745814 | A | AT | 0.60145 | 0.016001 | 0.0035605 | 194,174 | 6.99E-06 | 23.83672048 |
| rs939747826 | AT | A | 0.061186 | -0.03276 | 0.0071167 | 194,174 | 4.16E-06 | 23.9436059 |
| rs1230346 | C | T | 0.00026558 | -0.47502 | 0.10522 | 194,174 | 6.35E-06 | 23.26871725 |
| 6:131934981:CAAT:C | C | CAAT | 0.1526 | -0.026692 | 0.0048113 | 194,174 | 2.90E-08 | 35.7850688 |
| 6:138777931:G:GA | GA | G | 0.13502 | 0.023101 | 0.0051624 | 194,174 | 7.65E-06 | 24.20674905 |
| rs187791276 | A | G | 0.0026299 | -0.16347 | 0.034484 | 194,174 | 2.13E-06 | 27.22382787 |
| rs1187126 | T | A | 0.83845 | -0.020459 | 0.0046092 | 194,174 | 9.05E-06 | 22.0200745 |
| 6:43127082:T:TAG | TAG | T | 0.051312 | -0.033911 | 0.0076557 | 194,174 | 9.45E-06 | 21.74146318 |
| rs34311816 | T | C | 0.25509 | -0.018029 | 0.0039084 | 194,174 | 3.97E-06 | 23.9889239 |
| rs11974972 | G | A | 0.53767 | -0.017964 | 0.0033859 | 194,174 | 1.12E-07 | 31.1573315 |
| rs200872910 | C | CT | 0.38102 | -0.016985 | 0.0035268 | 194,174 | 1.47E-06 | 26.42598554 |
| rs1795403743 | G | GTGT | 0.0022114 | -0.18108 | 0.037998 | 194,174 | 1.88E-06 | 28.10129036 |
| rs2346264 | C | A | 0.78085 | 0.018242 | 0.0040917 | 194,174 | 8.27E-06 | 22.11668622 |
| rs170094 | T | C | 0.62352 | -0.015797 | 0.0035059 | 194,174 | 6.61E-06 | 22.75144674 |
| rs73102522 | T | C | 0.012901 | -0.070876 | 0.015534 | 194,174 | 5.06E-06 | 24.84589547 |
| rs1788249374 | TAGA | T | 0.54182 | -0.017006 | 0.0034556 | 194,174 | 8.60E-07 | 27.8852456 |
| rs12680355 | C | T | 0.13149 | 0.023839 | 0.0050314 | 194,174 | 2.16E-06 | 25.2067347 |
| rs1605128 | A | G | 0.21756 | -0.018529 | 0.004073 | 194,174 | 5.39E-06 | 22.69872289 |
| rs12056768 | G | T | 0.58028 | 0.020078 | 0.0034197 | 194,174 | 4.33E-09 | 38.13643172 |
| rs142170029 | C | T | 0.0071935 | -0.097896 | 0.021664 | 194,174 | 6.22E-06 | 26.5834172 |
| rs1306554825 | A | C | 0.0018401 | 0.19591 | 0.042803 | 194,174 | 4.72E-06 | 27.37994603 |
| rs6557679 | T | G | 0.2618 | -0.018919 | 0.0038574 | 194,174 | 9.37E-07 | 26.86684839 |
| 8:33692274:CT:C | C | CT | 0.074239 | 0.029547 | 0.0065293 | 194,174 | 6.03E-06 | 23.30378676 |
| rs2725674 | C | G | 0.83641 | -0.020299 | 0.0045773 | 194,174 | 9.23E-06 | 21.89731317 |
| rs28645375 | G | C | 0.01528 | 0.064479 | 0.014059 | 194,174 | 4.51E-06 | 24.29649565 |
| rs13284054 | C | T | 0.12517 | 0.024925 | 0.0052008 | 194,174 | 1.65E-06 | 26.42225908 |
| rs2033733945 | T | C | 0.816 | 0.020064 | 0.0044953 | 194,174 | 8.07E-06 | 23.47535252 |
| rs149815854 | G | A | 0.001765 | 0.20333 | 0.042361 | 194,174 | 1.59E-06 | 28.29178166 |
| rs1834744467 | G | GCGCCCACCACTA | 0.18057 | -0.020996 | 0.0044062 | 194,174 | 1.89E-06 | 25.33400461 |
| rs1381924 | G | A | 0.903543 | -0.027063 | 0.0057648 | 194,174 | 2.67E-06 | 24.79171082 |
| rs17467533 | C | G | 0.27973 | 0.017595 | 0.0037643 | 194,174 | 2.95E-06 | 24.2261106 |
| X:142235694:AGCT:A | A | AGCT | 0.012797 | -0.069668 | 0.015166 | 194,174 | 4.36E-06 | 23.81503285 |
| rs187457216 | T | C | 0.0018536 | 0.18904 | 0.042192 | 194,174 | 7.45E-06 | 25.67980463 |

**Supplementary Table S3. Baseline characteristics of FAH-DMU-DB participants.**

| Variables | 25(OH)D (nmol/L) | | | |  |
| --- | --- | --- | --- | --- | --- |
|  | <25 | 25-49.9 | 50-74.9 | >=75 | P |
| N | 259 | 534 | 579 | 1,070 |  |
| **Age** (years, mean ± SD) | 70.35±15.99 | 65.61±19.43 | 64.52±19.10 | 65.17±19.94 | <0.001 |
| **Gender** (%) |  |  |  |  | <0.001 |
| Male | 124 (47.88) | 217 (40.64) | 280 (48.36) | 611 (57.10) |  |
| Female | 135 (52.12) | 317 (59.36) | 299 (51.64) | 459 (42.90) |  |
| **Race** (%) |  |  |  |  | <0.001 |
| Han | 259 (100) | 534 (100) | 579 (100) | 1,070 (100) |  |
| **Marital status** (%) |  |  |  |  | <0.001 |
| Never married | 73 (28.19) | 167 (31.27) | 173 (29.88) | 294 (27.48) |  |
| Married | 186 (71.81) | 367 (68.73) | 406 (70.12) | 776 (72.52) |  |
| **BMI** (kg/m^2^, mean ± SD) | 28.61±6.56 | 29.15 ± 6.85 | 29.72±6.91 | 28.78±6.19 | <0.001 |
| **Diabetes** (%) |  |  |  |  | <0.001 |
| No | 231 (89.19) | 468 (87.64) | 491 (84.80) | 923 (86.26) |  |
| Yes | 28 (10.81) | 66 (12.36) | 88 (15.20) | 147 (13.74) |  |
| **Hypertension** (%) |  |  |  |  | <0.001 |
| No | 157 (60.62) | 358 (67.04) | 341 (58.89) | 689 (64.39) |  |
| Yes | 102 (39.38) | 176 (32.96) | 238 (41.11) | 381 (35.61) |  |
| **Drinking** (%) |  |  |  |  | <0.001 |
| No | 225 (86.87) | 462 (86.52) | 468 (80.83) | 825 (77.10) |  |
| Yes | 34 (13.13) | 72 (13.48) | 111 (19.17) | 245 (22.90) |  |
| **Smoking** (%) |  |  |  |  | <0.001 |
| Never | 192 (74.13) | 397 (74.34) | 416 (71.85) | 741 (69.25) |  |
| Former | 18 (6.95) | 37 (6.93) | 32 (5.53) | 62 (5.79) |  |
| Now | 49 (18.92) | 100 (18.73) | 131 (22.63) | 267 (24.95) |  |
| **OAB** (%) |  |  |  |  | <0.001 |
| No | 30 (11.58) | 128 (23.97) | 137 (23.66) | 269 (25.14) |  |
| Yes | 229 (88.42) | 406 (76.03) | 442 (76.34) | 801 (74.86) |  |
| **CVD** (%) |  |  |  |  | <0.001 |
| NO | 250 (96.53) | 519 (97.19) | 548 (94.65) | 1,039 (97.10) |  |
| YES | 9 (3.47) | 15 (2.81) | 31 (5.35) | 31 (2.90) |  |
| **Inflammatory biomarkers** (mean ± SD) |  |  |  |  |  |
| SII | 753.50±520.45 | 685.47± 601.66 | 659.77±609.82 | 615.00±722.29 | <0.001 |
| Neutrophils | 4.83±1.89 | 4.68±2.16 | 4.58±2.17 | 4.35±2.30 | <0.001 |
| WBC | 7.13±2.20 | 6.99±2.47 | 7.02±2.59 | 6.85±2.88 | <0.001 |
| NLR | 3.55±2.32 | 3.29±2.69 | 3.00±2.17 | 2.93±2.86 | <0.001 |

Mean ± SD for continuous variables, and P-value was calculated by weighted t-test. %, categorical variables, and P-value was calculated by weighted χ2 test.

SII, Systemic immune-inflammation index; WBC, White blood cell; NLR, neutrophil-to-lymphocyte ratio; OAB, Overactive bladder.

**Supplementary Table S4. Sensitivity analysis using multiple imputation for the association between 25(OH)D and the risk of OAB in NHANES.**

| Variables (%) | Model 1 OR (95%CI) | Model 2 OR (95%CI) | Model 3 OR (95%CI) |
| --- | --- | --- | --- |
| **Total** |  |  |  |
| Log_10_(25(OH)D) | 1.215 (1.05,1.406)0.009 | 0.796 (0.674,0.942)0.008 | 0.790 (0.665,0.938)0.007 |
| <25 | 1.0 | 1.0 | 1.0 |
| 25-49.9 | 0.749 (0.652,0.861)<0.001 | 0.834 (0.717,0.97)0.019 | 0.850 (0.729,0.991)0.038 |
| 50-74.9 | 0.687 (0.600,0.788)<0.001 | 0.784 (0.674,0.912)0.002 | 0.816 (0.699,0.952)0.010 |
| >75 | 0.870 (0.759,0.997)0.044 | 0.763 (0.653,0.890)0.001 | 0.772 (0.659,0.904)0.001 |
| **Female** |  |  |  |
| Log_10_(25(OH)D) | 0.916 (0.764,1.099)0.348 | 0.738 (0.598,0.911)0.005 | 0.726 (0.584,0.903)0.004 |
| <25 | 1.0 | 1.0 | 1.0 |
| 25-49.9 | 0.734 (0.614,0.876)0.001 | 0.832 (0.686,1.008)0.060 | 0.845 (0.694,1.027)0.091 |
| 50-74.9 | 0.655 (0.550,0.781)<0.001 | 0.770 (0.634,0.934)0.008 | 0.788 (0.647,0.959)0.018 |
| >75 | 0.749 (0.629,0.891)0.001 | 0.731 (0.601,0.890)0.002 | 0.734 (0.601,0.898)0.003 |
| **Male** |  |  |  |
| Log_10_(25(OH)D) | 1.760 (1.382,2.241)<0.001 | 0.973 (0.739,1.282)0.847 | 1.006 (0.757,1.337)0.966 |
| <25 | 1.0 | 1.0 | 1.0 |
| 25-49.9 | 0.826 (0.658,1.036)0.098 | 0.839 (0.655,1.074)0.163 | 0.869 (0.676,1.116)0.271 |
| 50-74.9 | 0.816 (0.654,1.018)0.072 | 0.803 (0.628,1.028)0.081 | 0.878 (0.683,1.128)0.308 |
| >75 | 1.089 (0.871,1.361)0.456 | 0.832 (0.646,1.071)0.153 | 0.871 (0.672,1.127)0.293 |

OR: Odds ratio; CI: Confidence interval.

Model 1: no covariates were adjusted.

Model 2: age, race, marital status, poverty income ratio, and education level were adjusted.

Model 3: age, race, marital status, poverty income ratio, education level, smoking, drinking, hypertension, diabetes, BMI, asthma, COPD, CVD, SII, vitamin D supplement use, season of blood collection and physical activity MET were adjusted

**Supplementary Table S5. Sensitivity analysis of the association between serum 25(OH)D levels and OAB using a more stringent definition (OABSS ≥ 4) in the NHANES cohort.**

| Variables (%) | Model1 OR (95%CI) | Model2 OR (95%CI) | Model3 OR (95%CI) |
| --- | --- | --- | --- |
| **Total** |  |  |  |
| Log_10_(25(OH)D) | 1.281 (1.009,1.627)0.042 | 0.684 (0.530,0.883)0.004 | 0.707 (0.547,0.913)0.008 |
| <25 | 1.0 | 1.0 | 1.0 |
| 25-49.9 | 0.670(0.542,0.829)<0.001 | 0.743 (0.593,0.933)0.010 | 0.766 (0.609,0.965)0.023 |
| 50-74.9 | 0.611(0.496,0.753)<0.001 | 0.676 (0.539,0.848)<0.001 | 0.722 (0.573,0.909)0.006 |
| >75 | 0.804 (0.654,0.989)0.040 | 0.642 (0.51,0.808)<0.001 | 0.666(0.527,0.841)<0.001 |
| **Female** |  |  |  |
| Log_10_(25(OH)D) | 0.927 (0.702,1.224)0.592 | 0.620 (0.457,0.842)0.002 | 0.632 (0.450,0.887)0.008 |
| <25 | 1.0 | 1.0 | 1.0 |
| 25-49.9 | 0.629(0.490,0.809)<0.001 | 0.676 (0.517,0.884)0.004 | 0.689 (0.524,0.906)0.008 |
| 50-74.9 | 0.586(0.458,0.750)<0.001 | 0.634 (0.485,0.830)<0.001 | 0.661 (0.501,0.872)0.003 |
| >75 | 0.668(0.523,0.852)<0.001 | 0.573 (0.437,0.752)<0.001 | 0.586(0.439,0.782)<0.001 |
| **Male** |  |  |  |
| Log_10_(25(OH)D) | 2.253(1.449,3.503)<0.001 | 0.940 (0.588,1.504)0.797 | 1.013 (0.611,1.680)0.960 |
| <25 | 1.0 | 1.0 | 1.0 |
| 25-49.9 | 0.904 (0.595,1.372)0.635 | 0.927 (0.599,1.434)0.733 | 0.970 (0.624,1.507)0.891 |
| 50-74.9 | 0.858 (0.570,1.292)0.463 | 0.804 (0.520,1.243)0.327 | 0.894 (0.574,1.393)0.622 |
| >75 | 1.240 (0.824,1.868)0.303 | 0.855 (0.549,1.330)0.486 | 0.918 (0.581,1.452)0.716 |

OR: Odds ratio; CI: Confidence interval.

Model 1: no covariates were adjusted.

Model 2: age, race, marital status, poverty income ratio, and education level were adjusted.

Model 3: age, race, marital status, poverty income ratio, education level, smoking, drinking, hypertension, diabetes, BMI, asthma, COPD, CVD, SII, vitamin D supplement use, season of blood collection and physical activity MET were adjusted.

**Supplementary Table S6. Association between 25(OH)D and the risk of overactive bladder in FAH-DMU-DB cohort.**

| Variables (%) | Model 1 OR (95%CI) | Model 2 OR (95%CI) |
| --- | --- | --- |
| **Total** |  |  |
| Log_10_(25(OH)D) | 0.516(0.363,0.733) < 0.001 | 0.465(0.310,0.699)<0.001 |
| <25 | 1.0 | 1.0 |
| 25-49.9 | 0.748(0.598,0.935)0.011 | 0.777(0.604,0.998)0.049 |
| 50-74.9 | 0.795(0.601,1.051)0.107 | 0.698(0.508,0.960)0.027 |
| >75 | 0.700(0.463,1.059)0.092 | 0.600(0.376,0.959)0.033 |
| **Female** |  |  |
| Log_10_(25(OH)D) | 0.385(0.235,0.629) < 0.001 | 0.373(0.216,0.643)<0.001 |
| <25 | 1.0 | 1.0 |
| 25-49.9 | 0.693(0.516,0.930)0.014 | 0.733(0.531,1.013)0.060 |
| 50-74.9 | 0.590(0.396,0.879)0.009 | 0.573(0.369,0.890)0.013 |
| >75 | 0.550(0.291,1.042)0.067 | 0.490(0.245,0.978)0.043 |
| **Male** |  |  |
| Log_10_(25(OH)D) | 0.679(0.411,1.123)0.132 | 0.752(0.416,1.359)0.346 |
| <25 | 1.0 | 1.0 |
| 25-49.9 | 0.803(0.569,1.134)0.213 | 0.882(0.591,1.316)0.538 |
| 50-74.9 | 0.936(0.625,1.402)0.748 | 0.914(0.572,1.463)0.709 |
| >75 | 0.906(0.523,1.573)0.727 | 0.954(0.501,1.817)0.886 |

OR: Odds ratio; CI: Confidence interval.

Model 1: no covariates were adjusted.

Model 2: age, race, marital status, smoking, drinking, hypertension, diabetes, BMI and CVD were adjusted.

**Supplementary Table S7**. **Association between Vitamin D supplement use and OAB**

| Variables (%) | Model 1 OR (95%CI) | Model 2 OR (95%CI) | Model 3 OR (95%CI) |
| --- | --- | --- | --- |
| **Total** |  |  |  |
| Vitamin D supplement use | |  |  |
| No | 1.0 | 1.0 | 1.0 |
| Yes | 1.265(1.187,1.348) <0.001 | 0.928(0.863,0.998)0.044 | 0.932(0.865,1.003)0.061 |
| **Female** |  |  |  |
| Vitamin D supplement use | |  |  |
| No | 1.0 | 1.0 | 1.0 |
| Yes | 1.092(1.004,1.187)0.039 | 0.874(0.795,0.961)0.006 | 0.894(0.811,0.984)0.023 |
| **Male** |  |  |  |
| Vitamin D supplement use | |  |  |
| No | 1.0 | 1.0 | 1.0 |
| Yes | 1.413(1.280,1.560) <0.001 | 1.031(0.921,1.154)0.597 | 1.007(0.898,1.129)0.910 |

OR: Odds ratio; CI: Confidence interval.

Model 1: no covariates were adjusted.

Model 2: age, race, marital status, poverty income ratio, and education level were adjusted.

Model 3: age, race, marital status, poverty income ratio, education level, smoking, drinking, hypertension, diabetes, BMI, asthma, COPD, CVD, SII, vitamin D supplement use, season of blood collection and physical activity MET were adjusted.

**Supplementary Table S8. Stratified two-piecewise regression results using 63.5 nmol/L as the inflection point for 25(OH)D**

| Subgroup | N | <63.5nmol/L(OR,95%CI) | ≥63.5nmol/L(OR,95%CI) | P for interaction |
| --- | --- | --- | --- | --- |
| **Age** |  |  |  | 0.098 |
| <50 | 6240 | 0.988 (0.983, 0.994) | 0.993 (0.987, 0.998) |  |
| 50-75 | 5096 | 0.989 (0.984, 0.994) | 0.999 (0.996, 1.002) |  |
| ≥75 | 1286 | 0.989 (0.978, 1.000) | 1.001 (0.997, 1.005) |  |
| **Race** |  |  |  | 0.736 |
| White | 5468 | 0.999 (0.991, 1.006) | 0.999 (0.996, 1.003) |  |
| Black | 2589 | 0.999 (0.993, 1.005) | 1.000 (0.995, 1.005) |  |
| Mexico | 1827 | 0.990 (0.981, 1.000) | 1.003 (0.995, 1.011) |  |
| Other | 2738 | 0.991 (0.983, 1.000) | 0.999 (0.993, 1.004) |  |
| **Education** |  |  |  | 0.084 |
| <High school | 1103 | 1.001 (0.990, 1.012) | 1.007 (1.000, 1.015) |  |
| High school | 4401 | 0.995 (0.990, 1.000) | 1.007 (1.003, 1.010) |  |
| >High school | 7118 | 0.989 (0.985, 0.994) | 1.005 (1.003, 1.008) |  |
| **Marital status** | |  |  | 0.232 |
| Never married | 5824 | 0.998 (0.993, 1.003) | 0.998 (0.995, 1.002) |  |
| Married | 6798 | 0.992 (0.986, 0.997) | 1.002 (0.999, 1.005) |  |
| **Poverty ratio** | |  |  | 0.110 |
| < 1.3 | 4851 | 0.994 (0.989, 1.000) | 1.006 (1.003, 1.010) |  |
| 1.3–3.5 | 4087 | 0.997 (0.991, 1.002) | 1.008 (1.005, 1.011) |  |
| > 3.5 | 3684 | 0.993 (0.985, 1.001) | 1.003 (0.999, 1.007) |  |
| **Diabetes** |  |  |  | 0.194 |
| No | 10873 | 0.992 (0.988, 0.995) | 1.005 (1.003, 1.008) |  |
| Yes | 1749 | 0.996 (0.989, 1.004) | 1.000 (0.996, 1.005) |  |
| **Hypertension** |  |  |  | 0.366 |
| No | 7878 | 0.990 (0.986, 0.995) | 1.002 (0.999, 1.005) |  |
| Yes | 4744 | 0.995 (0.990, 1.000) | 1.002 (0.999, 1.005) |  |
| **Drinking** |  |  |  | 0.091 |
| No | 6123 | 0.994 (0.989, 0.999) | 1.001 (0.998, 1.004) |  |
| Yes | 6499 | 0.996 (0.991, 1.002) | 0.998 (0.995, 1.002) |  |
| **Smoking** |  |  |  | 0.150 |
| Never | 2405 | 0.997 (0.989, 1.006) | 1.001 (0.997, 1.005) |  |
| Former | 2182 | 1.000 (0.992, 1.008) | 0.996 (0.990, 1.002) |  |
| Now | 8035 | 0.992 (0.987, 0.997) | 1.000 (0.997, 1.003) |  |
| **Asthma** |  |  |  | 0.724 |
| NO | 10480 | 0.993 (0.989, 0.997) | 1.005 (1.003, 1.007) |  |
| YES | 2142 | 0.991 (0.984, 0.999) | 1.004 (0.999, 1.008) |  |
| **CVD** |  |  |  | 0.769 |
| NO | 12271 | 0.995 (0.991, 0.999) | 1.000 (0.998, 1.002) |  |
| YES | 351 | 0.993 (0.975, 1.011) | 0.998 (0.988, 1.008) |  |

OR:Odds ratio; CI: Confidence interval.

**Supplementary Table S9. The results of two-piecewise logistic regression model.**

|  | Fully adjusted model | |
| --- | --- | --- |
| Outcome | Effect | P |
| **Model 1**  Fitting model by standard linear regression | 0.998 (0.997, 1.000) | 0.044 |
| **Model 2**  Fitting model by two-piecewise linear regression |  |  |
| Inflection point | 63.5 |  |
| <63.5 | 0.995 (0.991, 0.999) | 0.009 |
| ≥63.5 | 1.000 (0.998, 1.002) | 0.992 |
| p for likelihood test |  | 0.047 |

Fully adjusted model adjusts for age, race, marital status, poverty income ratio, education level, smoking, drinking, hypertension, diabetes, BMI, asthma, COPD, CVD, SII, vitamin D supplement use, season of blood collection and physical activity MET were adjusted.

**Supplementary Table S10. The associations between inflammatory biomarkers and overactive bladder in NHANES**

|  | Fully adjusted model | | |
| --- | --- | --- | --- |
| Variables | OR | 95%CI | P |
| SII | 1.001 | 1.0001, 1.0004 | 0.001 |
| Neutrophils | 1.068 | 1.0397, 1.0960 | 0.001 |
| WBC | 1.009 | 0.9925, 1.0254 | 0.291 |
| NLR | 1.054 | 1.0139, 1.0957 | 0.008 |

OR: Odds ratio; CI: Confidence interval; SII, Systemic immune-inflammation index; WBC, White blood cell; NLR, neutrophil-to-lymphocyte ratio.

Fully adjusted model adjusts for age, race, marital status, poverty income ratio, education level, smoking, drinking, hypertension, diabetes, BMI, asthma, COPD, CVD, SII, vitamin D supplement use, season of blood collection and physical activity MET were adjusted

**Supplementary Table S11. The associations between inflammatory biomarkers and overactive bladder in FAH-DMU-DB**

|  | Fully adjusted model | | |
| --- | --- | --- | --- |
| Variables | OR | 95%CI | P |
| SII | 1.0003 | 1.0001,1.0006 | <0.001 |
| Neutrophils | 0.9048 | 0.8610,0.9507 | <0.001 |
| WBC | 0.9641 | 0.9211,1.0091 | 0.1161 |
| NLR | 1.1706 | 1.1042,1.2409 | <0.001 |

OR: Odds ratio; CI: Confidence interval; SII, Systemic immune-inflammation index; WBC, White blood cell; NLR, neutrophil-to-lymphocyte ratio.

Fully adjusted model adjusts for age, race, marital status, smoking, drinking, hypertension, diabetes, BMI and CVD.

**Supplementary Table S12. Sensitivity analysis of MR.**

| Outcome | MR-PRESSO | | | MR-Egger | | | Cochran Q test | | |
| --- | --- | --- | --- | --- | --- | --- | --- | --- | --- |
|  | Causal estimate | SD | Global test *p* | intercept | SE | *p* | Q value | *p* | *I*^2^ |
| Unspecified urinary incontinence | -0.002 | 0.001 | 0.603 | 4.68E-05 | 3.90E-05 | 0.233 | 115.20 | 0.530 | 0.0% |
| Bladder problem | -0.0023 | 0.0011 | 0.714 | -7.92E-05 | 5.53E-05 | 0.155 | 97.997 | 0.898 | 0.0% |
